# Synchronization of visual perception within the human fovea

**DOI:** 10.1038/s41593-025-02011-3

**Published:** 2025-07-16

**Authors:** Annalisa Bucci, Marc Büttner, Niklas Domdei, Federica B. Rosselli, Matej Znidaric, Julian Bartram, Tobias Gänswein, Roland Diggelmann, Martina De Gennaro, Cameron S. Cowan, Wolf Harmening, Andreas Hierlemann, Botond Roska, Felix Franke

**Affiliations:** 1 Institute of Molecular and Clinical Ophthalmology Basel (IOB), Basel, Switzerland; 2 University of Basel, Faculty of Science, Basel, Switzerland; 3 Department of Biosystems Science and Engineering (D-BSSE), Eidgenössische Technische Hochschule (ETH) Zürich, Basel, Switzerland; 4 Department of Ophthalmology, Rheinische Friedrich-Wilhelms-Universität Bonn, Bonn, Germany

**Keywords:** Retina, Retina, Neurophysiology

## Abstract

The human brain constructs a model of the world by processing sensory signals with distinct temporal characteristics that may differ in generation and transmission speed within a single sensory modality. To perceive simultaneous events as occurring at the same time, the brain must synchronize this sensory information, yet the mechanisms underlying such synchronization remain unclear. By combining human neural recordings, behavioral measurements and modeling, we show that in the human visual system, this process begins in the fovea centralis, the retinal region used for reading and recognizing faces. Reaction times to foveal single-cone photostimulation were similar across the central visual field, although visual information from neighboring foveal cones travels along axons of highly different lengths. From direct measurements of action potential propagation speeds, axon diameters and lengths in the human fovea centralis, we found that longer foveal axons have larger diameters and increased propagation speeds. We conclude that the human brain orchestrates axonal conduction speeds of unmyelinated axons in the retina to synchronize the arrival times of sensory signals. These results suggest a previously unknown mechanism by which the human brain synchronizes perception.

## Abstract (web summary)

Combining behavioral data, electrophysiology and modeling, the authors show that the human brain synchronizes visual signals by adjusting axonal conduction speed in the retina, revealing a previously unknown mechanism for precise perceptual timing.

## Main

**Fig. 1:**
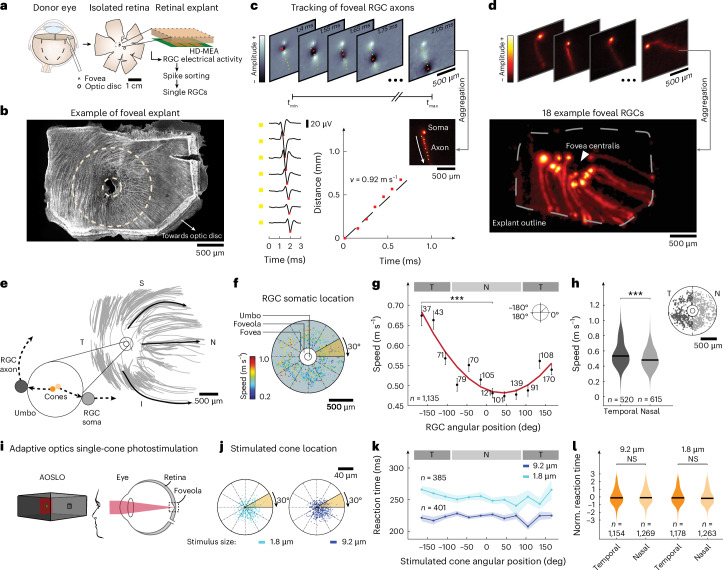
Action potential propagation speeds systematically vary around the human fovea centralis*.* **a**, Sample preparation (cross, fovea; ellipse, optic disc). **b**, Beta-III-tubulin-immunolabeled foveal explant post-HD-MEA recording. The hole in the central fovea resulted from tissue removal after HD-MEA recordings. Small circle, foveola; large circle, fovea. **c**, Top: successive frames of AP waveform video of an RGC axon, 1.4–2.05 ms postinitiation. Color—waveform amplitude. Bottom left: average AP waveform at increasing distances from the soma (yellow squares). Red square, waveform trough. Bottom right: linear regression (dashed line) of travel time versus distance. **d**, Top: electrical images of representative foveal RGCs. Bottom: composite image of 18 representative RGCs. **e**, Top: estimated RGC axon trajectories for one preparation. Three axons are accentuated (black arrows). Small circle, umbo (magnified below); large circle, foveola. Inset: cone-to-RGC connectivity in the umbo with adjacent cones (orange discs) and target RGCs (gray discs). **f**, Somatic location of 1135 RGCs (11 explants). Color—axonal propagation speeds; radial lines, 30° bins. **g**, Axonal propagation speed (mean ± s.e.m.) of RGCs within the angular bins from **f**. Red line, best-fitting sinusoid. Significant difference (Kruskal–Wallis test, ***P < 0.001) between −165° (0.67 ± 0.02 m s^−1^) and 15° (0.48 ± 0.01 m s^−1^). T and N regions are indicated by gray bars. **h**, Comparison of axonal propagation speeds between T (dark gray) and N (light gray) foveal RGCs. Median speeds—T, 0.53 m s^−1^ and N, 0.48 m s^−1^ (two-sided Wilcoxon rank-sum test, ***P < 0.001). Data from **g** replotted by spatial bin. **i**, Schematic of light stimulation with AOSLO. **j**, Locations of light stimulation in the foveola (using AOSLO) relative to the CDC in one participant. Radial lines, 30° bins. **k**, Reaction times (mean ± s.e.m.) for trials in **j**. No significant differences between angular bins (Kruskal–Wallis test; smallest *P* values—large spot, *P* = 0.25; small spot, *P* = 0.99). T and N regions are indicated by gray bars. **l**, Normalized reaction times in all seven participants (two-sided Wilcoxon rank-sum test; large spot—NS, *P* = 0.34 and small spot—NS, *P* = 0.13). S, superior; N, nasal; I, inferior, T, temporal; Norm., normalized. Source data

**Extended Data Fig. 1:**
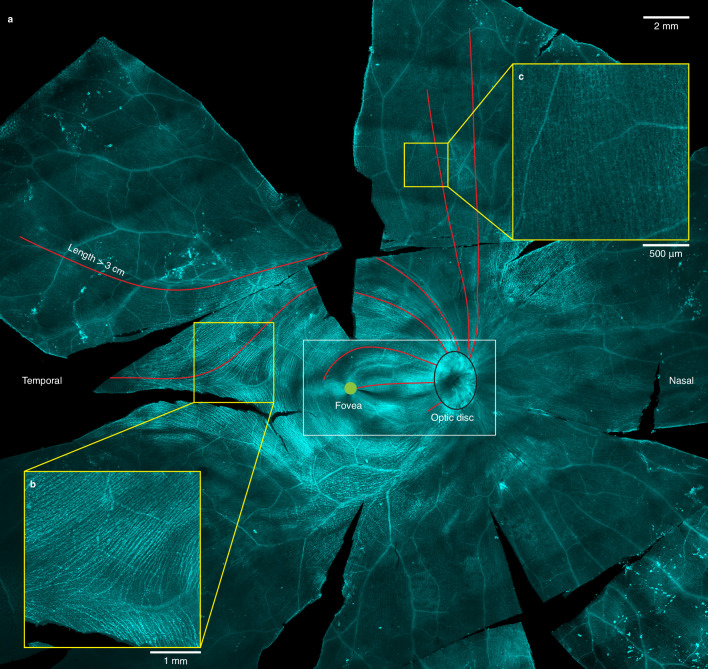
Axon bundles in the human retinal nerve fiber layer. **a**, Human whole-mount retina immunolabeled for axon bundles with beta-III tubulin (cyan). White rectangle: Region depicted in Extended Data Fig. 2a. Red lines: examples of axonal trajectories. **b**,**c**, Enlarged depictions of the respective regions marked in **a**. Source data

**Extended Data Fig. 2:**
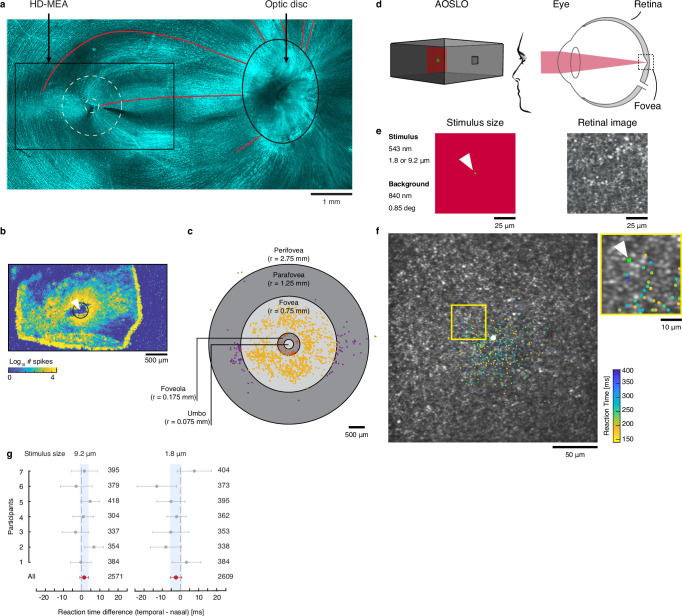
Electrical activity and reaction times in the human fovea centralis. **a**, Human whole-mount retina immunolabeled for axon bundles with beta-III tubulin (cyan). The image shows the area marked in Extended Data Fig. 1a. Black rectangle: outline of HD-MEA active array. White dashed circles: foveola (small) and fovea (large). **b**, Spontaneous electrical activity of an example ex vivo human foveal explant recorded with an HD-MEA (different preparation than in **a**). Color codes for the number of spikes recorded at each electrode within a 30 s recording window. The pixel position encodes the electrode position. White arrowhead: center of the fovea; black circle: outline of the foveola. **c**, Positions of RGC somas relative to the fovea centralis, with cells in specific regions indicated in color: foveola (orange, *N* = 37), fovea (yellow, *N* = 1135), parafovea (purple, *N* = 108) and perifovea (green, *N* = 5). The umbo, at the center, lacks RGCs. Concentric rings indicate subregion boundaries. **d**, Schematic of light stimulation with an AOSLO. **e**, Left: illustration (to scale) of the light stimulus. Red square: raster of the AOSLO. The white arrowhead indicates the stimulus. Green square: stimulus. Right: retinal image of one participant. **f**, Left: individual stimulus locations (colored points) depicted on top of one participant’s retinal image. White dot: location of the cone density centroid. The point color encodes the reaction time at each location. Right: enlarged depiction of the region marked by a yellow square on the left. The white arrowhead points to a sketch of a 1.8 × 1.8 µm large stimulus (green square). **g**, Statistical analysis for the reaction-time data of each of the seven participants (gray) and the combined data (bottom, red). Error bars: mean ± 90% confidence intervals. Blue region: confidence interval of the combined data. Vertical dotted line: 0 ms effect size is contained in all but two confidence intervals. Source data

To construct a temporally consistent model of the world, the brain needs to integrate information from simultaneous events across sensory modalities with different temporal characteristics, such as varying signal generation or propagation speeds. Even within a single sensory modality, information from different parts of the sensory space may arrive at different times. The precise relative timing of incoming information in higher brain areas can be highly relevant. In the auditory cortex, the brain can extract behaviorally relevant information about the location of a sound source from the relative timing of arriving action potentials from both ears with a resolution of less than 1 µs (refs. ^1,2^). When integrating information from both eyes, timing differences below 10 ms are relevant for depth perception^3,4^. The human eye has a diameter of 25 mm, and the retina extends over an area of approximately 1,100 mm^2^ (ref. ^5^). Locally, the retinal circuitry processes visual signals within each small image patch synchronously. This process unfolds across the entire retina, resulting in the generation of action potentials by retinal ganglion cells (RGCs). To travel from the eye to the brain, these action potentials must reach the optic disc, where the optic nerve begins and exits the eyeball. RGCs extend their axons to the brain, but the intraretinal lengths of these axons depend on the specific location of the RGCs within the retina, ranging from a few hundred micrometers near the optic disc to more than 3 cm in the periphery (Extended Data Figs. 1 and 2a). Even for RGCs that convey electrical signals from immediately adjacent photoreceptors within the fovea centralis, axonal lengths can differ substantially. In the umbo, the very center of the fovea, photoreceptor axons connect radially outward to displaced bipolar cells within the foveal shoulder, which, in turn, connect to RGCs arranged in a ring-like structure around the umbo^6^. As neighboring photoreceptors can connect to RGCs on opposite sides of this ring, proximity in visual space does not imply proximity in anatomical space (Fig. 1e). However, human participants do not perceive temporal dispersion of signals from different parts of the visual field, which raises the question of how compensation for different travel distances is achieved.

Here we combined anatomical modeling of intraretinal axon trajectories, electrophysiological recordings of RGC action potentials in human retinae—including the fovea centralis*—*measurements of axonal diameters, and behavioral assessments of human visual reaction times to investigate whether RGCs compensate for differences in travel distance by adjusting axonal propagation speeds. Our findings reveal that intraretinal axon diameter and conduction speed increase with axon length and partially compensate for differences in travel distance across the retina. In the fovea, this compensation reduces the temporal dispersion of coeval retinal signals at the optic disc to less than 2.5 ms, thereby helping to preserve temporal fidelity in visual perception despite substantial anatomical disparities in axonal length.

## Results

### Axonal speed depends on RGC soma location in the human fovea

**Extended Data Fig. 3:**
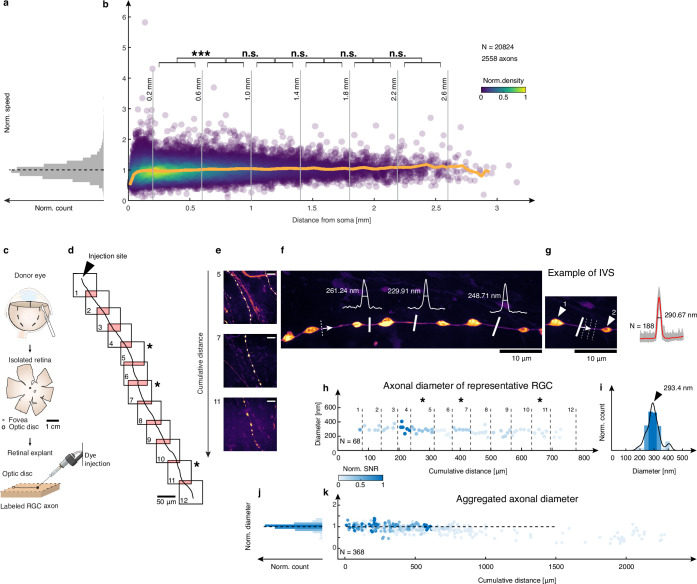
Action potential propagation speed and axon diameter along RGC axons. **a**, Histogram of normalized, local action potential propagation speed measurements. **b**, Density-colored scatter plot of normalized propagation speed values (20,824 speed measurements along 2,558 axons from 34 retinal explants). Each point represents the local action potential speed measured along short (usually <35 µm) axonal segments. Orange line: mean normalized speed. Black brackets (top): statistical comparisons between consecutive 0.4 mm bins, starting 0.2 mm away from the soma. Although the first test was significant (two-sided Wilcoxon rank-sum test, ***P < 0.001), the effect size was small (~5% speed increase; first bin: 0.98 ± 0.23; second bin: 1.03 ± 0.24; mean ± s.d.). **c**, Schematic of the RGC axon labeling procedure by injection of a current. Panel **c** is created with BioRender.com. **d**, Example of a labeled RGC axon across 12 FOVs. **e**, Three example FOVs (5, 7, 11 in **d**) at increasing distance from injection site. Scale bar: 10 µm. Images from one axon, representative of seven axons from three retinae. **f**, RGC axon segment in one field of view (~400 µm distance from injection site). White: intensity profiles and FWHM estimates measured at three sampling points along an axon segment. Representative of the axon shown in **d** and **e**. **g**, Same field of view as **f**. Left: example of two consecutive varicosities (white triangles). Intensity profile is measured between the varicosities. Right: diameter of the IVS estimated as FWHM of the average intensity profile (red) between two consecutive varicosities. **h**, Successive IVS diameter measurements as a function of distance from the injection site. Points are color-coded according to normSNR of the field of view. Data are from the same axon shown in **d**–**g**, with multiple IVS measurements per field of view. **i**, Weighted histogram showing the distribution of measured diameters for the same axon shown in **d**–**h**. Solid line: kernel density estimate. N refers to the number of IVS diameter measurements (individual points shown in **h**). **j**, Average normalized diameter at increasing distance from the injection site. Histogram includes 368 pooled IVS diameter measurements from seven RGC axons across three retinae. **k**, normSNR-colored scatter plot of normalized IVS diameter values (same data as in **j**). Dashed line: unity. Source data

To measure the time necessary for action potentials of foveal RGCs to reach the optic disc at high spatial and temporal resolution, we recorded the spiking activity of RGCs in the human fovea by means of complementary metal-oxide semiconductor (CMOS)-based, planar high-density microelectrode arrays (HD-MEAs)^7–10^. We dissected donor eyes to isolate the entire retina (Fig. 1a), subsequently resected retinal explants approximately 3 mm × 2 mm in size, containing the fovea, and placed them RGC-side down onto the microelectrode array. The preparation enabled simultaneous recordings of RGC action potentials from foveola, fovea, parafovea and a small portion of the perifovea (Extended Data Fig. 2c). After the recordings, we immunolabeled the RGC axon bundles to ascertain the presence of the fovea centralis within the resected explants (Fig. 1b). We identified the electrical activity of individual neurons through offline spike sorting^11^ of the electrical recordings and reconstructed the electrical image (average electrical waveforms of action potentials per electrode) for each neuron across the entire chip (~26,000 electrodes) at a sampling rate of 20 kHz (Fig. 1c). Superimposing a subset of the electrical images revealed the location of the fovea centralis on the chip and the ring-like arrangement of RGC somas (Fig. 1d). We visualized the electrical images of individual RGCs as videos at a frame rate of 20 kHz. In each video, action potentials became visible as voltage deflections traveling across the electrodes of the HD-MEA surface (Fig. 1c and Supplementary Videos 1 and 2). We tracked 1,135 individual foveal RGC axons over distances of up to 1.7 mm (10 donors, 11 explants). The propagation speed remained largely unchanged along the axons, except near the soma (Extended Data Fig. 3a,b). We then calculated the axonal propagation speeds through linear regression of the traveled distance versus travel time for each RGC while ignoring the initial 200 µm close to the soma (representative RGC shown in Fig. 1c). We registered axonal trajectories of different preparations in a reference coordinate system by aligning the location of the fovea centralis and the orientation between all resected retinal explants. This procedure revealed the axonal wiring pattern around the fovea centralis, which closely resembled the pattern visible in immunolabeled RGC axon bundles (Fig. 1b). Figure 1f shows the somatic locations of all tracked foveal RGCs. To quantify the dependence of the axonal action potential propagation speed on the RGC soma location within the ring around the fovea centralis, we binned the angular location of the RGC somas in 12 angular bins, each spanning 30° (Fig. 1f) and compared the average speeds within the bins (Fig. 1g). This approach revealed a strong dependence of the action potential propagation speed on the angular location of foveal RGCs. Specifically, action potentials of RGCs situated temporal to the umbo (that is, away from the optic disc) propagated more than 40% faster than those of RGCs situated nasal to the umbo (that is, closer to the optic disc).

### Uniform reaction times to foveal single-cone stimulation

**Extended Data Fig. 4:**
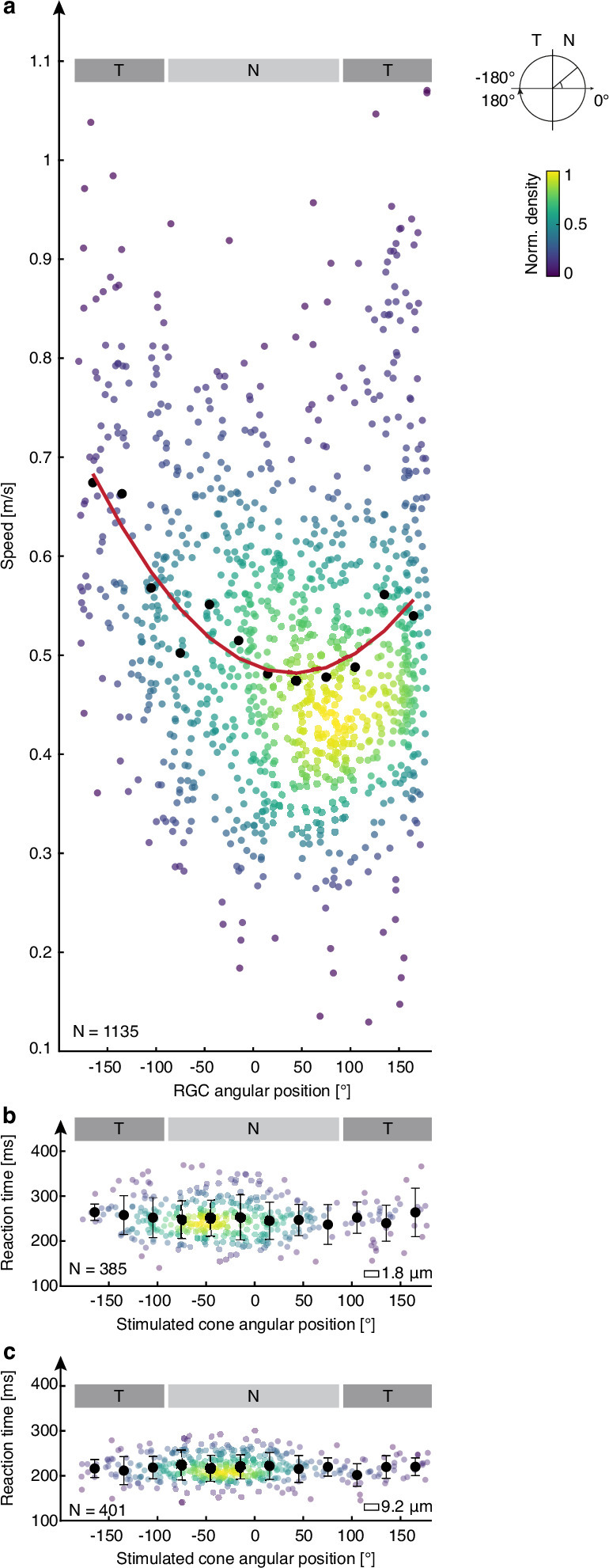
Angular dependence of axonal propagation speed and reaction times. **a**, Axonal propagation speed of RGCs as a function of angular position. Colored dots: individual RGCs colored according to normalized density (min–max scaling); binned averages (black dots, 30° bins) and best-fitting sinusoid (red line) as in Fig. 1g. Temporal (T) and nasal (N) regions are indicated by gray bars. **b**,**c**, Reaction times for a single participant as a function of stimulated cone angular position for small (**b**, 1.8 μm) and large (**c**, 9.2 μm) stimuli. Same data as Fig. 1k. Colored dots: individual trials colored by normalized density. Binned averages (black dots, 30° bins, ± s.e.m.) as in Fig. 1k. Temporal (T) and nasal (N) regions as in **a**. Number of trials is indicated. Source data

Within the retina, foveal RGC axons originating in locations temporal to the fovea centralis are substantially longer than those originating on the nasal side and extending directly toward the optic disc (Fig. 1b and Extended Data Fig. 2a). We investigated whether the observed increase in action potential propagation speed of these axons may compensate for their greater length. We refer to this as the ‘equal travel time hypothesis’, suggesting a mechanism that synchronizes action potential arrival times at the optic disc for action potentials initiated simultaneously across the fovea centralis. In contrast, under an ‘equal propagation speed hypothesis’, action potentials from RGCs with longer axons would feature delays in arrival times at the optic disc, which would potentially increase human reaction times to localized visual stimuli. Previous studies have shown an increase in human reaction times to localized visual stimulation with greater eccentricity from the fovea^12^. To test whether human reaction times to localized foveal stimulation align with the ‘equal travel time hypothesis’, and to ensure precise and selective stimulation of the densely packed cones within the fovea centralis, we conducted a series of psychophysical experiments using adaptive optics scanning light ophthalmoscopy (AOSLO)^13^. We measured the temporal dispersion of human reaction times in response to brief flashes of small squares of light (1.8 µm × 1.8 µm or 9.2 µm × 9.2 µm) presented in the umbo (Fig. 1i and Extended Data Fig. 2d–f). Reaction times were quantified by measuring the time interval between the onset of the light flash and the pressing of a button by seven participants. Responses of one participant are depicted in Fig. 1j,k (mean reaction time to 1.8 µm squares = 250 ± 42 ms and to 9.2 µm squares = 218 ± 28 ms), while results aggregated from all participants are presented in Fig. 1l. We used the cone density centroid (CDC), which represents the topographical center of the foveal cone mosaic^14^, as the center of the fovea. Similar to our method of sampling the angular positions of RGCs around the fovea centralis, we assessed the angular positions of the stimulation locations in the umbo by grouping them into 12 angular bins, each spanning 30°, relative to the CDC (Fig. 1j,k and Extended Data Fig. 4b,c). We normalized each participant’s data by subtracting their mean reaction time and dividing by their respective standard deviation. Subsequently, we grouped the stimulation locations into two regions relative to the CDC—temporal and nasal. We observed no significant difference (Fig. 1l). In fact, the data excluded with high confidence that temporal reaction times were more than 1.0 ms and 5.6 ms faster than nasal reaction times for the large and small squares, respectively (Extended Data Fig. 2g). This finding aligns with the ‘equal travel time hypothesis’.

### Axonal propagation speeds increase with eccentricity

**Extended Data Fig. 5:**
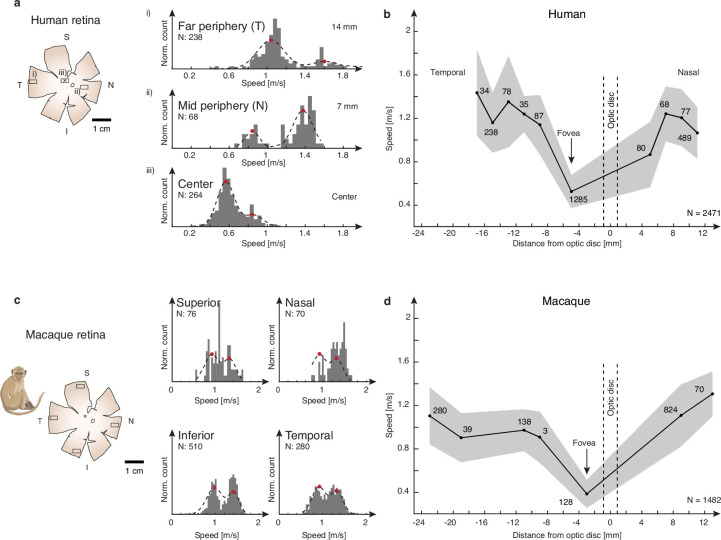
Dependence of axonal propagation speed on eccentricity in human and macaque retinae. **a**, Histograms of normalized action potential speed from three different locations in the human retina (schematic on the left) showing bimodal distributions. Dashed line: fit of Gaussian mixture model with two components; red dots: position of the peaks. Far periphery (i, peaks: 1.04 m s^−1^ and 1.60 m s^−1^, *N* = 238), mid periphery (ii, peaks: 0.84 m s^−1^ and 1.38 m s^−1^, *N* = 68) and center (iii, peaks: 0.57 m s^−1^ and 0.84 m s^−1^, *N* = 264). **b**, Mean action potential speeds in human retinal explants along the naso-temporal axis by distance from the optic disc; temporal (negative *x* axis) versus nasal (positive *x* axis). The numbers indicate the RGC count; shaded region: mean ± s.d. Dashed vertical lines mark optic disc boundaries. **c**, Histograms of normalized action potential speeds in macaque retina (four explants from the far periphery). S: superior (peaks: 0.92 ms^−1^ and 1.31 m s^−1^), N: nasal (0.93 m s^−1^ and 1.31 m s^−1^), I: inferior (0.98 m s^−1^ and 1.40 m s^−1^), T: temporal (0.92 m s^−1^ and 1.31 m s^−1^, *N* = 280). Dashed line: fit of Gaussian mixture model with two components; red dots: position of the peaks. Illustration in panel **c** is created with BioRender.com. **d**, Same as **b** but for macaque retina. Source data

To understand whether axonal action potential propagation speeds also vary across different regions of the peripheral retina, which exhibit large disparities in axonal lengths, we measured propagation speeds at different locations and eccentricities. To this end, we measured RGC action potentials across human and nonhuman primate (*Macaca fascicularis*) retinae with the same method as described above but with explants isolated at different retinal locations not including the fovea. We recorded signals from 16 peripheral human retinal explants, isolated along the naso-temporal axis from seven donors, which yielded a total of 1,186 tracked human peripheral RGC axons (*v* = 1.13 ± 0.30 m s^−1^, *v*_max_ = 2.31 m s^−1^, *v*_min_ = 0.16 m s^−1^; max tracked length = 3.06 mm). Extended Data Fig. 5b illustrates the average axonal propagation speed along the naso-temporal axis of the human retina. The propagation speed measured in foveal explants was lowest (indicated by the arrow in Extended Data Fig. 5b, *n* = 1,285) and increased as the eccentricity from the fovea increased. In the far periphery (>10 mm distance from the foveal pit, >30° eccentricity), the increase in speed was less pronounced. We obtained similar results for macaque retinae, which exhibited propagation speeds of similar magnitude and dependence on the retinal location. In macaques, we recorded the electrical activity of 128 foveal (four explants) and 1,354 peripheral (eight explants) RGCs along the naso-temporal axis (Extended Data Fig. 5d). In macaques, we also recorded the electrical activity at four equi-eccentric but radially distant locations (superior, inferior, temporal and nasal) in the far periphery. Although the distributions of propagation speeds exhibited some differences among the four locations, the average speeds were of similar magnitude (Extended Data Fig. 5c).

### Identifying RGC types from responses to light stimulation

**Fig. 2:**
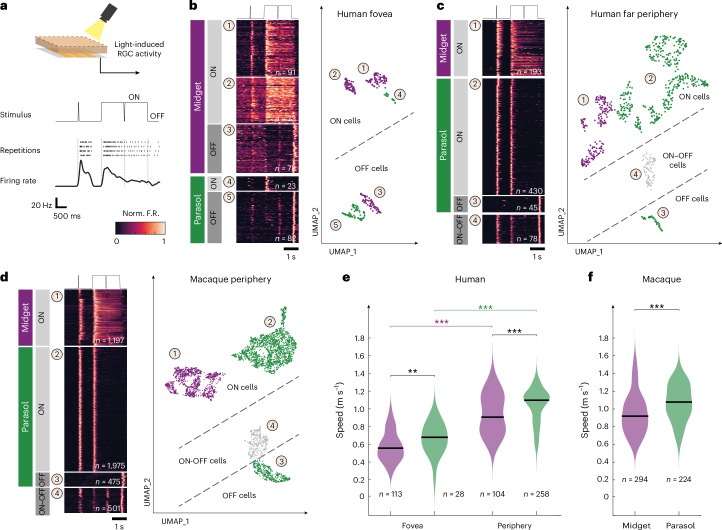
Functional cell typing reveals the dependence of axonal propagation speed on eccentricity in midget and parasol cells and in human and macaque retinae. **a**, From top to bottom: schematic of HD-MEA recordings with light stimulation. ‘ON–OFF’ light stimulus (contrast over time); raster plot of the spiking response of a representative ON midget RGC. Each row represents a single stimulus presentation, and each small vertical dash represents a spike; average firing rate over trials is depicted below. F.R., firing rate. **b**–**d**, Clustering of light-induced RGC responses to identify functional cell types of the human fovea (**b**), human periphery (**c**) and macaque periphery (**d**). Left: normalized firing rates (averaged over trials) of all RGCs depicted as rows in response to the stimulus depicted above. Cells were grouped by clusters (number in circle). Labels on the left indicate the putative cell type for groups of clusters. Right: functional clusters (UMAP projection). UMAP coordinates in **b**–**d** were rotated to reflect similarity in the cluster structure. Each dot represents an RGC. The colors correspond to the cell type (left). The numbers in the circle indicate the cluster number. Dashed lines are visual aides that separate cell types of different response polarities. **e**, Distributions of action potential speeds in midget (purple) and parasol (green) cells in human fovea and periphery. Foveal (midget, 0.56 ± 0.17 m s^−1^; parasol, 0.68 ± 0.17 m s^−1^; median ± s.d.); peripheral (midget, 0.91 ± 0.22 m s^−1^; parasol, 1.10 ± 0.20 m s^−1^; median ± s.d.). Two-sided Wilcoxon rank-sum test—foveal, **P < 0.01; peripheral, ***P < 0.001. Intratype comparison shows lower speeds in the fovea than in the periphery. Group medians are indicated. **f**, Same as **e** but for macaque periphery (median speed midget, 0.92 ± 0.24 m s^−1^; median speed parasol, 1.08 ± 0.21 m s^−1^; two-sided Wilcoxon rank-sum test, ***P < 0.001). Source data

The primate retina features two main types of RGCs, midget and parasol cells, which constitute over 90% of all primate RGCs^15–17^. Previous work in the macaque retina has shown that peripheral midget cells have lower action potential propagation speeds (~0.8 m s^−1^) than parasol cells (~1.2 m s^−1^)^18^. Furthermore, the relative number of midget and parasol cells depends on the retinal location^19^. Approximately 90% of RGCs in the fovea are midget cells, whereas this percentage drops to approximately 40–45% in the periphery^20^. Hence, the elevated speed of axonal action potential propagation in peripheral regions may result from the sampling of a greater proportion of parasol cells compared to the fovea. We investigated whether we could distinguish the two cell type populations in our data. Extended Data Fig. 5a shows the distributions of propagation speeds measured in three explants originating from three different locations along the naso-temporal axis in the human retina, from the far periphery (~14 mm from the optic disc), the mid periphery (~7 mm from the optic disc), and the center (explant centered on the fovea, about 4.7 mm from the optic disc). The distributions were bimodal, and both modes shifted toward lower propagation speeds with decreasing eccentricity. To show that the two distribution peaks indeed corresponded to the two cell types, we measured the light responses of a subset of the recorded RGCs to full-field light stimulation (Fig. 2a). Midget and parasol cells have different roles in primate vision and correspondingly exhibit different response behaviors upon stimulation with steps and brief flashes of light; these responses can be used to identify cell types^16,21–25^. Midget cells show longer sustained responses to steps in the average brightness compared to parasol cells, which produce more transient responses^16,24,25^. We projected a 2-s-long dark screen, interrupted by a 16-ms-long bright flash, followed by a step to a 2-s-long bright screen, interrupted by a 16-ms-long dark flash (Fig. 2a). We then represented the neural response of each RGC as a high-dimensional feature vector and used a dimensionality reduction technique to project the high-dimensional dataset onto two dimensions (Uniform Manifold Approximation and Projection (UMAP)^26^; Fig. 2b–d). In addition to and independent of the dimensionality reduction, we clustered the RGC data to identify groups of RGCs featuring similar light-evoked responses, and which likely belonged to the same cell type. We performed this analysis independently for the three different datasets from human fovea (Fig. 2b), human periphery (Fig. 2c) and macaque periphery (Fig. 2d). We then labeled the groups as midget or parasol cells based on the similarity of the average response within each group and the known response behavior of midget and parasol cells. This way, we classified a total of 5,241 RGCs. In each of the three datasets, we could identify response behaviors that can be expected from the main primate RGC types—cells showing increased activity upon positive contrast changes (ON cells), negative contrast changes (OFF cells) and cells that responded to both changes (ON–OFF cells). The ON–OFF cell cluster was absent in recordings from the fovea centralis. The absence of ON–OFF cells aligns with prior results indicating that small bistratified cells, characterized by ON–OFF response behavior, are less prevalent in the fovea^20^. Additionally, we identified cells that exhibited transient responses to contrast changes, a characteristic trait of parasol cells, as well as cells that displayed sustained activity in response to such contrast changes, a typical behavior observed in midget cells^16,24^.

### Midget and parasol cell axon speeds rise with eccentricity

We measured both the propagation speeds and light responses of 1,021 RGCs (human fovea, 141; human periphery, 362; macaque periphery, 518). For these cells, we analyzed the action potential propagation speed as a function of cell type (Fig. 2e,f). Across both cell types, axonal action potential propagation speeds were greater in the periphery than in the fovea, and axons of midget cells propagated action potentials at lower speeds than axons of parasol cells. Specifically, in the human foveal region, midget and parasol cells featured median propagation speeds of 0.6 ± 0.2 m s^−1^ and 0.7 ± 0.2 m s^−1^, respectively. In both human and macaque periphery, midget cells exhibited median axonal action potential propagation speeds of 0.9 ± 0.2 m s^−1^—slower than parasol cells—which showed speeds of 1.1 ± 0.2 m s^−1^. Midget cells in the periphery demonstrated higher axonal action potential propagation speeds than parasol cells in the fovea, underscoring the complex interplay between cell types and retinal locations in determining axonal speed. Within a single retinal location, we found a strong association between functional cell type and action potential propagation speed, suggesting that—at given retinal locations—action potential propagation speed alone is a good indicator to distinguish midget from parasol cells. However, for a reliable speed-based classification, it is necessary to record RGCs at the same retinal location, as different locations feature vastly different speed distributions, which would confound the classification.

### A model of the axonal trajectories across the human retina

**Fig. 3:**
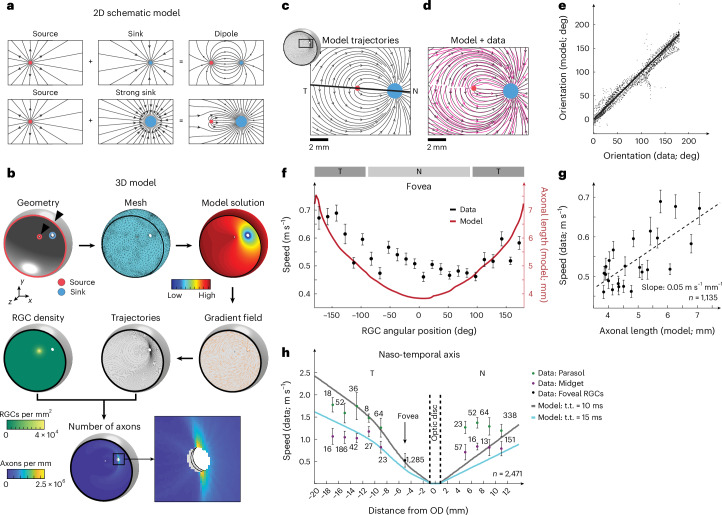
A model based on Laplace’s equations demonstrates that axonal propagation speed correlates to axonal length. **a**, Examples of solutions to Laplace’s equations and how they linearly combine to yield new solutions. Black lines—field lines of the underlying potential, that is, trajectories along the potential gradient. Red dot, source; blue dot, sink. **b**, A 3D model of axonal trajectories in the human retina. Top row: left—eye geometry with sources (red circles, highlighted by black triangles) and sink (blue circle); middle—generated mesh for the numerical solution of Laplace’s equation; right—scalar field solution representing the chemical concentration diffusing from the ora serrata and fovea to the OD. Middle row (right to left): right—orientation of the concentration gradient, guiding axonal growth (orange lines); middle—example axonal trajectories following the gradient (black lines); left—RGC density across the retina used to calculate axon numbers per location. Bottom left: axon counts per location. Inset: zoomed view of the OD (small black square). **c**, Modeled axonal trajectories (black lines) in the foveal region corresponding to Extended Data Fig. 6c. Solid black line, fovea-to-OD axis. **d**, Superposition of the modeled trajectories from **c** with the estimated trajectories from the whole mount in Extended Data Fig. 6c. **e**, Comparison of the modeled and estimated trajectories for the region in **d**. Solid line, unity; each point represents local orientations of small image patches. **f**, Foveal speed data from Fig. 1g binned every 15° (mean ± s.e.m., black, axis on the left) overlaid with model axonal length (solid red line, axis on the right). T and N regions are indicated by gray bars. **g**, Same speed data as in **f** (mean ± s.e.m.) plotted against model axonal length with linear regression fit (dashed line). **h**, Speed data (mean ± s.d.) from Extended Data Fig. 5b by RGC type (midget, purple; parasol, green) and for foveal RGCs (black); numbers, RGCs per bin; dashed vertical lines, OD boundaries; solid lines, speeds corresponding to 100% compensation for t.t. of 10 ms (gray) and 15 ms (cyan). OD, optic disc; t.t., travel times. Source data

**Extended Data Fig. 6:**
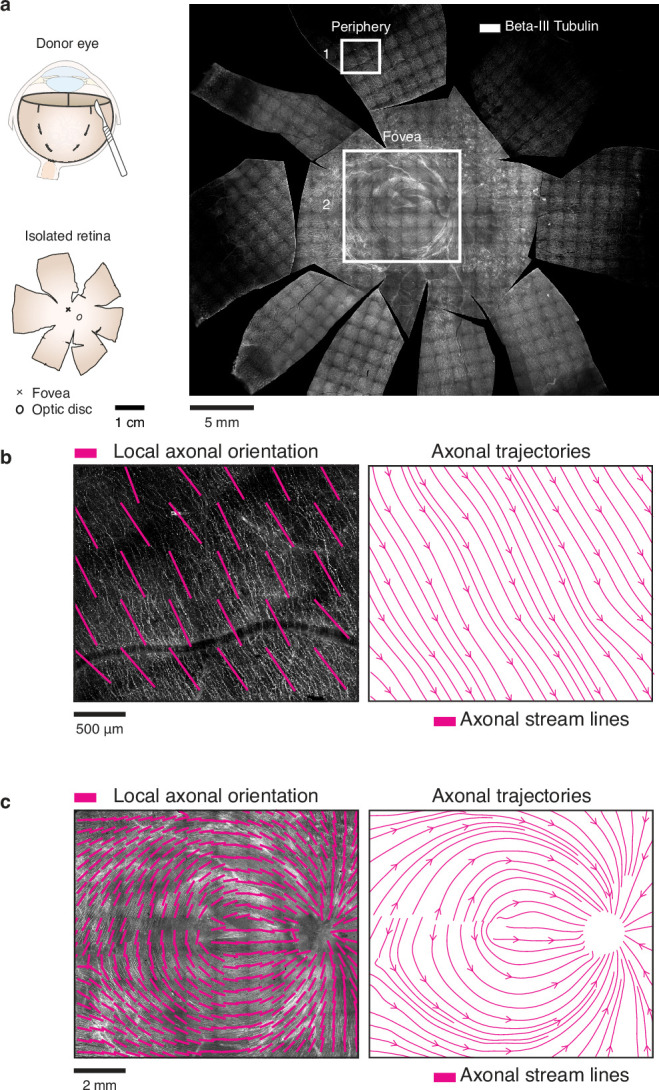
Estimation of axonal trajectories in whole-mount human retinae. **a**, Schematic of tissue preparation (left) and human whole-mount retina immunolabeling for axon bundles with beta-III tubulin (right). The two white rectangles depict the regions (1, periphery; 2, fovea) highlighted in **b** and **c**. **b**, Estimation of axonal trajectories from the immunolabeled whole-mount shown in **a**. Left, zoom into the image in **a** at a peripheral location superimposed with the estimated local axonal orientation (short magenta line segments). Right, estimated axonal trajectories (magenta lines) at that location. **c**, Same as **b**, but for a region centered on the fovea. Source data

So far, we measured axonal propagation speeds as a function of RGC somatic location and cell type. However, to understand to what degree the observed speed difference compensates for differences in axonal length, we needed to correlate the measured speed with the intraretinal axonal length. To this end, we developed a mathematical model that described the precise trajectories of all RGC axons across the entire human retina. As a starting point, we used the observation that the pattern of axonal trajectories around the human fovea that was visible in our whole-mount images (Extended Data Figs. 1a, 2a and 6a) resembled field lines of magnetic fields, or streamlines of fluid flow under a laminar-flow regime (Fig. 3a). The field lines are solutions to Laplace’s equation, which is a second order partial differential equation. Laplace’s equation describes many physical phenomena, including diffusion. Under steady-state conditions, the local concentration of a diffusing chemical does not change. Consequently, the amount of the chemical that enters a certain spatial compartment must be exactly equal to the amount that leaves this compartment—Δ*c* = 0, where *c* is the concentration and Δ is the Laplace operator or spatial derivative. When axons grow, they establish their trajectories by following gradients of specific chemicals with their growth cones^27^. These chemicals are often distributed by diffusion; therefore, it is plausible to also use Laplace’s equation, which describes diffusion processes, to describe axonal trajectories. Laplace’s equation is linear and therefore abides by the superposition principle. Figure 3a (top) illustrates the superposition of a sink and a source (both solutions to Laplace’s equation), which yields a dipole (a third solution). If we modify this example by making the sink stronger than the source, the resulting pattern of field lines changes and strongly resembles the axonal trajectories around the human fovea (Fig. 3a, bottom). Motivated by this observation, we developed a 3D model of the geometry of the human eye and solved Laplace’s equation for the semi-spherical geometry of the human retina. We placed a weak, but spatially extended source at the location of the fovea, a stronger sink at the location of the optic disc, and another circular source at the rim of the retina (that is, at the ora serrata; Fig. 3b), motivated by observations of ring-like concentration gradients of molecules at the ora serrata in the developing eye that guide axon growth^28^. Apart from the geometry, only five parameters specified the entire model—the relative strengths of the two sources and the sink, the spatial extent of the foveal source and the diffusivity of the retinal tissue. Our model yielded a concentration gradient of a chemical, created at the fovea and ora serrata, and absorbed at the optic disc (Fig. 3b, top right). If an RGC growth cone started at any location in the retina and followed this gradient, it would reach the optic disc along a trajectory determined by the field lines. Thus, the resulting trajectory was the corresponding field line. To test whether our model accurately described axonal trajectories in the human retina, we estimated the trajectories of axonal bundles across the human retina in immunolabeled whole-mount retinal images (Extended Data Fig. 6) by an automated procedure (Extended Data Fig. 6b,c). We then fitted the five parameters of our model to the extracted trajectories in the central area containing fovea and optic disc and compared the model to the data (Fig. 3c–e). Despite the low number of parameters, the model described the axonal trajectories qualitatively and quantitatively well (fovea 1, *R*^2^ = 0.91; fovea 2, *R*^2^ = 0.95).

**Extended Data Fig. 7:**
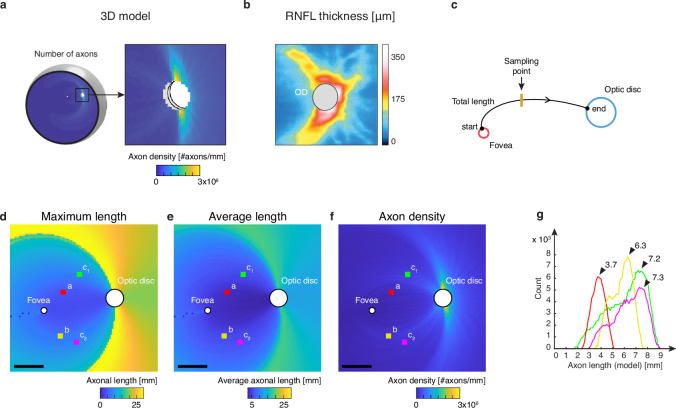
Model prediction of retinal nerve fiber layer thickness. **a**, Number of axons at each location within the retina. Same as in Fig. 3b. Right: zoomed view of the region containing the optic disc (white hole in the center). **b**, Thickness of the RNFL of one of the authors measured with OCT. Gray ellipsoid: optic disc. **c**, Schematic showing example of an axonal trajectory passing a sampling point. Yellow rectangle: sampling point to measure axonal diameter. **d**–**f**, Region of the retina containing fovea and optic disc. Color indicates different statistics of the model axons traversing each pixel. Colored squares mark locations where TEM measurements (Fig. 4a) were conducted. Scale bar: 2 mm. **d**, Maximum length. **e**, Average length. **f**, Number of model axons (axon density). **g**, Histograms of the lengths of all axons that traverse each of the four locations marked in **d**–**f**. Colors are the same as in **d**–**f**. Arrowheads over histogram maxima indicate the *x* position (length) of the maxima. Source data

We verified the model’s validity by predicting the thickness of the retinal nerve fiber layer (RNFL). The RNFL is the innermost retinal layer and consists of unmyelinated RGC axons. The more axons pass through a location in the retina, the thicker the RNFL is, and the RNFL thickness can be assessed in vivo by optical coherence tomography (OCT)^29^. We modeled the RGC density as a function of retinal location based on measurements of primate RGC densities^30,31^. We arranged the axons along the field lines of our 3D model, determined the respective axon densities, and counted how many RGC axons passed through that location for each location within the RNFL. The resulting axon densities agreed qualitatively with measurements of the RNFL thickness in healthy participants (Extended Data Fig. 7b).

### Propagation speed compensates for retinal axonal length

We then used the model to correlate axonal action potential propagation speeds with intraretinal axonal length with the aim of estimating the intraretinal travel time of action potentials from RGC somas to the optic disc. In the following, ‘axonal length’ refers to the intraretinal axonal length as defined by the model.

In the ring-like structure around the fovea centralis (radius = 0.25 mm), axonal lengths ranged from a minimum of 3.8 mm on the nasal side to a maximum of 7.5 mm on the temporal side (Fig. 3f). These values demonstrate that under the ‘equal propagation speed hypothesis’ (where all action potentials travel at the same speed), action potentials starting on the temporal side of the fovea centralis would take nearly twice as long to reach the optic disc as action potentials starting on the nasal side.

**Extended Data Fig. 8:**
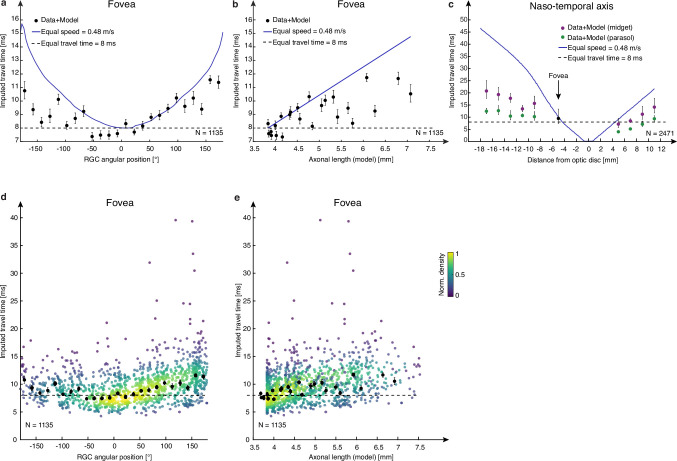
Imputed travel times from soma to optic disc for different axonal lengths and different length-to-speed relationships. **a**, Imputed travel times (mean ± s.e.m.) as a function of RGC angular position. Dashed black line: equal travel time (8 ms). **b**, Imputed travel times (mean ± s.e.m.) as a function of model axonal length. **c**, Imputed travel times (mean ± s.e.m.) along the naso-temporal axis as a function of distance from the optic disc. **a**–**c**, Blue solid line: travel time assuming equal propagation speed (0.48 m s^−1^). Dashed black line: equal travel time (8 ms). **d**,**e**, Scatter plots of individual imputed travel times as a function of the angular position of foveal RGCs (**d**) and model axonal length (**e**), color coded by normalized density (min–max scaling). Binned averages (black dots, 30° bins, ±s.e.m.) are the same as those shown in **a** and **b**, respectively. Dashed black line: equal travel time (8 ms). Source data

Under this hypothesis, a speed of 0.48 m s^−1^ resulted in ~15 ms travel time for temporally located RGCs versus ~8 ms for nasally located RGCs (Extended Data Fig. 8a). However, the measured action potential propagation speeds correlated with the modeled axonal lengths (Fig. 3f,g) so that the difference between minimal and maximal travel times (‘temporal dispersion’) was substantially reduced (Extended Data Fig. 8a,b). A correlation between propagation speed and eccentricity also existed in the periphery for midget and parasol cells (Fig. 3h); that is, longer axons showed higher propagation speeds. For each of the locations where we measured propagation speeds, we used the model to calculate axonal lengths and, under the ‘equal travel time hypothesis’, the necessary speeds to achieve equal travel times. For parasol cells, 10 ms travel time corresponded well to the measured speeds temporally to the optic disc, whereas for midget cells, a value of 15 ms travel time was more appropriate. Generally, the measured differences in speeds only partially compensated for the differences in axonal length (solid lines in Fig. 3h and Extended Data Fig. 8c).

### Axonal thickness determines axonal propagation speed

**Fig. 4:**
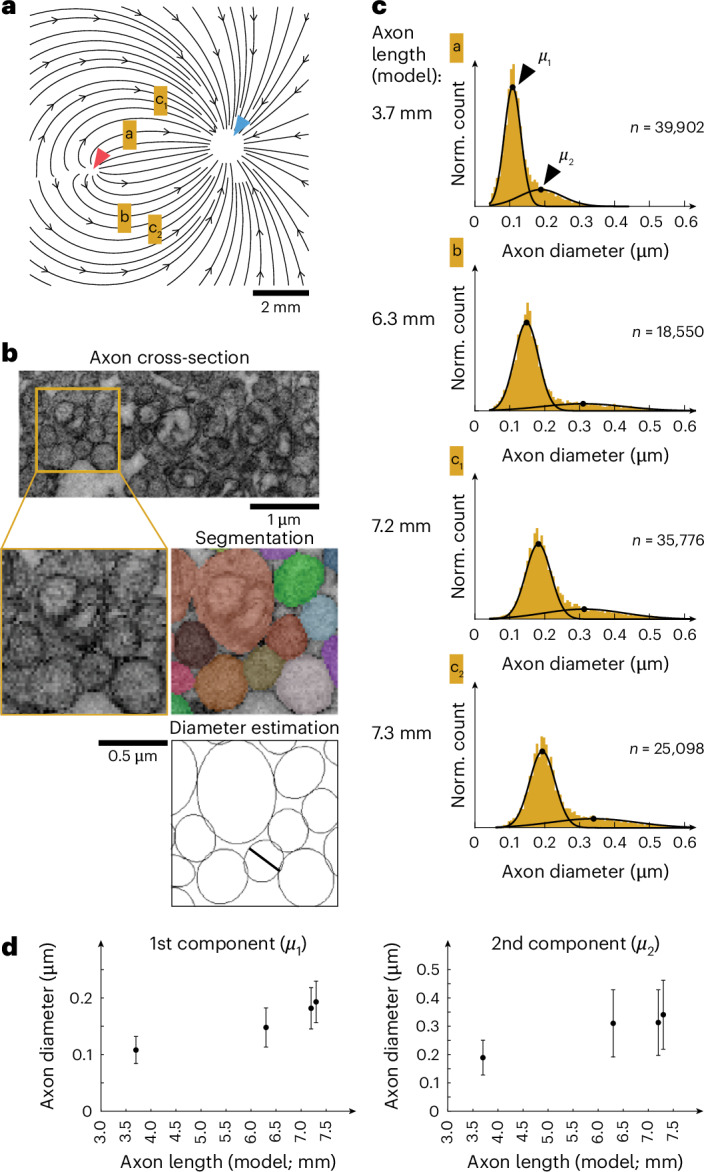
RGC axon diameters increase with intraretinal axonal length. All results were obtained from a single donor. **a**, RGC axon sampling locations (lowercase letters a, b, c_1_, c_2_) overlaid with model axonal trajectories. Red triangle, fovea; blue triangle, optic disc. **b**, Top: cropped region from a TEM image of a cross-section of RNFL axons. Middle: a magnified view of the TEM image (left) and corresponding segmentation (right; colors indicate different axons). Bottom: outlines of segmented axons; black line indicates the minor axis of an ellipse fit to one axon outline. The image shown is from one of four sampling locations in a single human retina and is representative of the segmentation and analysis performed at all four locations. **c**, Histograms of RGC axon diameters at four different locations sampled from one human retina. Model axonal length for the four locations marked on the left of each histogram. The numbers indicate the number of estimated RGC axon diameters per location; black lines, fit of Gaussian mixture model with two components; black dots, mean value of each component (*μ*_1_ and *μ*_2_). **d**, Mean ± s.d. of the two Gaussian components fit in **c** versus model axonal length (Extended Data Fig. 7d–g). Means and s.d. obtained by fitting Gaussian mixture models to the distributions shown in **c**. Source data

A main factor determining axonal propagation speed in unmyelinated axons is their thickness or diameter, with larger diameters reducing axial resistance and thereby enhancing conduction speeds. This relationship scales proportionally with the square root of the axon diameter^32^. We examined the influence of unmyelinated RGC axon thickness on the speed of action potential propagation. Previous findings have highlighted a positive correlation between retinal eccentricity and axonal diameter, with axons of more eccentric RGCs being thicker^33^. Notably, peripheral primate parasol cells exhibit greater propagation speeds^34^ and thicker axons than midget cells^35^. To assess whether variations in axonal thickness could account for the observed differences in action potential propagation speed around the human fovea centralis, we employed transmission electron microscopy (TEM) to measure RGC axonal diameters at four different retinal locations (TEM ‘sampling locations’) around the fovea centralis. We first isolated explants (~1 mm^2^ in size) from the central region of postmortem human retinae as shown in Fig. 4a. We then processed these samples into 70 nm thick cross-sections of resin-embedded tissue, which were subsequently imaged with TEM at ×8,500 magnification. Each image covered patches of RNFL ranging in size from 100 to 200 µm. Postimaging, we segmented the images to distinguish the RGC axon cross-sections and delineate their outlines (Fig. 4b, top). Diameter estimates were derived by fitting ellipses to these outlines and measuring their minor axes (Fig. 4b, bottom), which resulted in more than 110k human retinal axon diameters. This analysis revealed bimodal diameter distributions at the four different locations, which we fitted using Gaussian mixture models with two components (Fig. 4c). The first component represented axons of small diameter and high numerical abundance, whereas the second component represented axons with larger diameters, small abundance and larger variability in diameter. For each of the four TEM sampling locations, we calculated the corresponding axonal length using our model and then related the model axon length to the average axon diameters. To calculate axonal length at a TEM sampling location, we sampled 170k streamlines across the retina, counting those passing the sampling location within 100 μm distance. Each streamline was weighted by RGC density at its origin (Fig. 3b and Extended Data Fig. 7c). For each location, we then calculated a histogram of the counted, weighted streamline lengths to determine the number of axons with specific lengths passing the location. These histograms (Extended Data Fig. 7g) revealed that the axons with the most prevalent length at each location originated from the foveal rim. We defined the prevalent length (mode of the histogram) as the intraretinal length at the sampling location. Axon diameters positively correlated with intraretinal axon length, and the average diameter of axons increased by ~80% when comparing lengths of 3.7–7.3 mm (Fig. 4d). These results were in qualitative agreement with our action potential propagation speed measurements (Fig. 3g). Using dye injections, we labeled individual human RGC axons up to 2.4 mm length and measured their diameters optically. While axon diameters strongly varied within a few micrometers, the diameters seemed to be comparably constant over longer distances (Extended Data Fig. 3c–k).

## Discussion

The region of the human retina responsible for high-acuity vision, the umbo at the center of the fovea, lacks RGCs and their axons^36,37^, which loop around this area. The low intraretinal axonal conduction speed and the large differences in axonal lengths could lead, in the brain, to a substantial temporal dispersion of arrival times of coeval (that is, synchronously evoked) action potentials.

**Extended Data Fig. 9:**
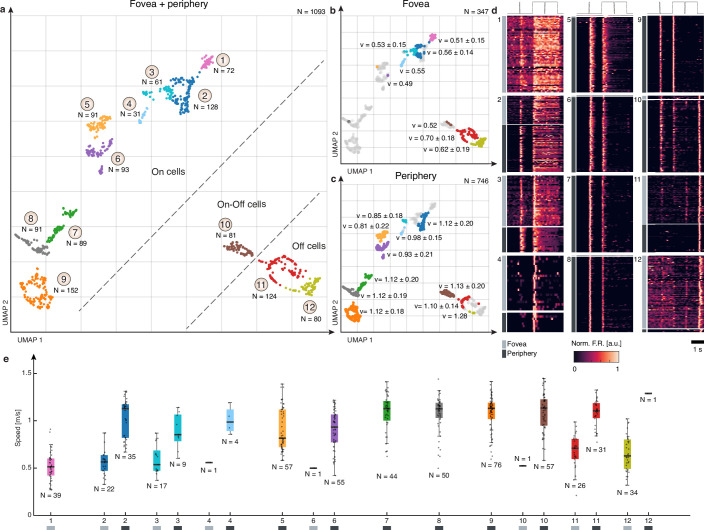
Clustering analysis of RGC responses of human fovea and periphery and their action potential propagation speeds. **a**, UMAP projection of both foveal and peripheral RGC data, clustered into 12 distinct color-coded groups based on their functional properties (*N* = 1093 cells). The dashed line separates On, On–Off and Off cells. **b**,**c**, Same UMAP projection as in **a** (gray dots) with cells from the fovea (**b**, *N* = 347) and periphery (**c**, *N* = 746) marked by colors. Same color scheme as in **a**. Median propagation speeds (*v*, m s^−1^) and standard deviations are reported for each cluster. Standard deviations are not given for three clusters, which contained only a single cell. **d**, Normalized firing rates (averaged over trials and coded by color) of all RGCs depicted in **a** as rows in response to the stimulus depicted above. Each panel represents a single cluster, with foveal RGC responses shown at the top and peripheral responses below. Light and dark gray shading at the left indicates foveal (light gray) and peripheral (dark gray) cells in each cluster. **e**, Box plots of action potential propagation speeds (individual data points overlaid). The number of samples for each box plot is indicated*N*, and the colors correspond to the clusters shown in panels **a**–**c**. Box plots show extrema, 25th and 75th percentiles and median. Some clusters show paired data for fovea and periphery, while others represent only one region. Color shading below the cluster numbers indicates foveal (light gray) and peripheral (dark gray) data for each cluster. Source data

By combining multiple experimental approaches with modeling of the RNFL, we related human reaction times, axonal conduction speeds, and intraretinal RGC axonal lengths and diameters with soma location and functional RGC type across the human retina. We showed that, in humans and macaques, intraretinal axonal length was positively correlated with axonal diameter and action potential propagation speed. This correlation reduced the temporal dispersion of coeval action potentials at the optic disc and contributed to compensating for different travel distances. For coeval action potentials evoked in the human fovea, this compensation reduced the temporal dispersion at the optic disc to <2.5 ms, which was consistent with our behavioral measurements of human reaction times, where we found an average reaction time difference of less than 1.0 ms between the temporal and nasal regions of the fovea centralis for the larger stimuli. We found a similar correlation between axonal length and action potential propagation speed in the peripheral retina. The propagation speed differences between foveal and peripheral RGCs were consistent across all functional clusters and were not specific for individual cell types (Extended Data Fig. 9). A previous study did not find any correlation between propagation speed and axon length in rabbit RGCs^10^. The mechanism described here may, therefore, be specific to primates or animals with larger eyes, which exhibit larger differences in intraretinal axonal lengths.

**Extended Data Fig. 10:**
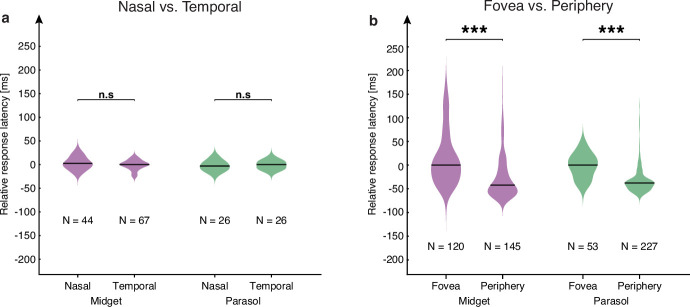
Estimation of RGC response latency in human fovea and periphery. **a**, Relative response latencies of midget (purple) and parasol (green) cells in response to the ‘ON–OFF’ light stimulus in human nasal and temporal fovea. Response latencies are relative to the median absolute response latency of the temporal RGCs. Foveal midget (nasal: 2.8 ± 13.6 ms; temporal: 0.0 ± 10.5 ms; median ± s.d.; two-sided Wilcoxon rank-sum test: n.s., *P* = 0.24); foveal parasol (nasal: −3.0 ± 11.1 ms; temporal: 0.0 ± 9.2 ms; median ± s.d.; two-sample *t* test: n.s., *P* = 0.52). **b**, Relative response latencies of midget (purple) and parasol (green) cells in human fovea versus periphery. For both midget and parasol cells, response latencies are relative to the median absolute foveal response latency; peripheral midget (−42.5 ± 47.3 ms; median ± s.d.); peripheral parasol (−38.0 ± 27.9 ms; median ± s.d.). Two-sided Wilcoxon rank-sum test: midget ***P < 0.001, parasol ***P < 0.001. The standard deviations of the foveal data in **a** and **b** are different, because a more robust analysis was used to estimate the latencies in **a**. In **a**, the latencies were estimated by the cross-correlation of the firing rate to a template. This method was not applicable to the data in **b** (Methods). Latencies in **b** were estimated by the peak of the firing rate after a light stimulus. For details, see Methods. Source data

In the fovea and for midget cells in the periphery, the measured speeds did not fully compensate for the increased axonal lengths (Fig. 3h and Extended Data Fig. 8). To synchronize the arrival times of signals at the brain, different compensation mechanisms could be at play. Upstream of action potential propagation, response latency of RGCs could change with eccentricity; that is, RGCs with longer axons could respond more quickly to light stimulation. For example, the response latency of foveal RGCs could be increased by the increased length of their photoreceptor axons^38^. Indeed, it has been shown that peripheral midget cells respond around 30 ms faster than foveal midget cells^39^. We confirmed this finding in our data and found the same difference in response latency for parasol cells (Extended Data Fig. 10b). We also analyzed whether temporal foveal RGCs responded faster than nasal foveal RGCs, but this was not the case (Extended Data Fig. 10a). Our action potential speed measurements together with the model axonal length can be used to calculate the imputed travel time, that is, the expected action potential travel time from the soma to the optic disc for each RGC. We superimposed these travel times with travel times predicted by the ‘equal travel time’ and ‘equal propagation speed’ hypotheses (Extended Data Fig. 8). The results indicated that for cells in the foveal region and for midget cells in the periphery our data only support partial compensation of axonal length by propagation speed, whereas for parasol cells in the periphery, the results were more aligned with the ‘equal travel time hypothesis’ (Extended Data Fig. 8c). Together with the differences in RGC response latency, our findings suggest that for a single retina-wide light flash peripheral action potentials may arrive at the optic disc before foveal action potentials. However, RGC response latencies strongly depend on stimulus parameters like contrast and size^40,41^, and by integrating stimuli over a larger area, the larger peripheral RGCs may feature shortened latencies to large stimuli with respect to foveal RGCs. The above considerations may, therefore, depend on stimulus parameters. Previous studies have shown a contrast-dependent increase in human reaction times to small visual stimuli with increasing eccentricity^12^.

Our TEM data suggest that for a length increase of ~100% (3.7–7.3 mm; Fig. 4d), the axon diameter increases by 80% (0.11–0.19 µm). According to cable theory, if action potential propagation speed is proportional to the square root of the axonal diameter, this diameter increase should yield a 34% speed increase. Our foveal action potential speed measurements indeed indicated that for a length increase from 4 mm to 7 mm (75% increase; Fig. 3g), the speed increased by ~30% (0.50–0.65 m s^−1^). However, the diameter and speed data were in agreement with a $\nu\propto \surd{d}$ relationship; the observed speed increase was insufficient to fully compensate for the larger axonal length. In other words, under the ‘equal travel time hypothesis’, the longer axons would require a much larger speed increase of nearly 100% and a corresponding ~290% increase in diameter than what we measured.

Downstream of the intraretinal action potential propagation, the travel time in the optic nerve and the signal integration in the brain could also contribute to synchronizing the arriving signals. The brain could, for example, use relative timing information of pairs of RGCs^42^. In the optic nerve, there is a strong relationship between axon diameter and propagation speed^43,44^, and the axon diameter is correlated to the retinal location of the RGCs^45^. It has been speculated that propagation speed adjustments in the optic nerve could help to tune arrival times in the brain^46^, or that propagation speed could be shaped by constraints on information transmission^44^.

In summary, RGC response latency, intraretinal travel times, travel times in the optic nerve, and integration in the brain will all contribute to synchronize the visual signals, and this synchronization mechanism likely depends on stimulus parameters and retinal eccentricity.

Previous studies have shown that the average axon diameter is positively correlated with eccentricity^35,47,48^, and that foveal RGC axons feature smaller average diameters than RGC axons in the periphery^33^. Indirect measurements of axonal conduction speed, based on patterned electroretinograms in humans, indicated a positive correlation between speed and eccentricity^49^. However, the two main RGC types in the primate retina, midget and parasol cells^15,16^, have different axonal diameters^48^ and different axonal propagation speeds^18,34,50^, and their relative abundance depends on retinal location^19^. Therefore, the relative numbers of sampled cell types can be a confounding factor when interpreting differences in average axon diameters and conduction speeds at different retinal locations. Furthermore, RGC axons can follow nonstraight trajectories from soma to optic disc so that the intraretinal length of these axons strongly depends on retinal location, which is not accounted for when axons are grouped by eccentricity (Fig. 1b,e). Therefore, we directly measured axonal conduction speeds and functional cell types of individual RGCs, and—by using our model—we correlated the results with retinal location, axonal length and diameter.

Our findings highlight the existence of intricate synchronization mechanisms early in the signaling cascade of the human visual system. Moreover, we provide evidence for the hypothesis that the conduction speeds of even unmyelinated axons in the human brain are modulated to synchronize perception.

## Methods

### Human retinal tissue

All samples were anonymized. These procedures complied with the principles of the Declaration of Helsinki and were approved by the local ethics committee (Ethikkommission Nordwest-und Zentralschweiz). Human eyes were obtained from multi-organ donors with no documented history of eye diseases. The donors, encompassing both sexes, ranged in age from 30 to 80 years. Enucleations were performed by the Augenklinik Basel in collaboration with the University Hospital of Basel.

### Human retinal explants and electrophysiological setup

Post enucleation, the corneal tissue was excised for transplantation purposes, and the vitreous humor was carefully removed following radial incisions on the eye bulbs. Critically, we minimized the time between clamping of the eye’s central artery, which interrupted blood supply, and the subsequent immersion of the eyes in pre-oxygenated (95% O_2_ and 5% CO_2_) Ames’ medium (Sigma-Aldrich, A4034). The enucleated eyes, or more specifically, the eyecups, were rapidly transported to our laboratory, maintained in an actively oxygenated environment (PanGas AG HiQ; Minican, 800002225), consistently under 20 min. This rapid processing was critical for maintaining tissue viability for subsequent electrophysiological recordings. Retinal explants were then isolated and flattened by relaxing cuts under dim red-light conditions in oxygenated Ames’ medium at room temperature. Explants, approximately 6 mm^2^ in size, were placed flat on the CMOS HD-MEAs^7^ with the RGC layer facing the electrodes. To enhance signal-to-noise ratio (SNR), the explants were affixed to the electrodes using a transparent cell culture membrane (Transwell‑Clear; Corning, 3450), pressed against the photoreceptor layer. To ensure precision and to avoid damaging the retinal circuitry, the membrane was lowered under constant visual inspection using a micromanipulator (Thorlabs, MBT616D/M) connected to a custom device for maintaining the membrane flat. For electrophysiological recordings, we maintained the explants in a constantly perfused oxygenated Ames’ medium and temperature-controlled environment. The Ames’ medium was warmed to 37 °C by a temperature controller (Multi Channel Systems MCS GmbH, TC01/02) and delivered at a flow rate of 6 ml min^−1^ using a peristaltic pump (Darwin Microfluidics, BT100-1L). The used medium was removed by suction through a centralized vacuum line connected to a siphon system. This setup ensured the viability of the retinal tissue, allowing for extended recording durations of up to 20 h.

### Nonhuman primate retinal explant preparation

All procedures performed on the animals were approved by the Comité Régional d’Ethique en Matière d’Expérimentation Animale de Strasbourg and registered with the following numbers: APAFIS 5716_2016061714424948_v6 (28 August 2018), APAFIS 32591_2021072914362019_v5 (3 April 2022) and APAFIS 27357-2020092811266511_v2 (28 December 2020). We used retinal explants of 15 healthy adult cynomolgus macaques (*M. fascicularis*). These animals were housed and monitored at the Simian Laboratory Europe (SILABE) in compliance with the European Directive (2010/63/EU). Retinal explants were sourced from macaques, courtesy of our collaborators. All animals were killed for different research projects, which involved the treatment of some of the eyes by subretinal injection but did not make use of the complete retinal tissue. The enucleation process was conducted under deep terminal anesthesia with ongoing monitoring. It is imperative to perform enucleations before killing to maximally preserve vascularized tissues and prevent cellular damage due to oxygen deprivation. The anesthesia and analgesia protocols guaranteed that the animals remained unconscious and free from pain throughout the entire procedure, up until the point of killing. The enucleation protocol included the following steps: animals were fasted the night before the procedure, then sedated with ketamine (10 mg kg^−1^, intramuscularly) and transported to the preparation room. A venous catheter was inserted into the saphenous vein, followed by an intravenous injection of Propofol (Propovet, 5–10 mg kg^−1^) through the catheter. The animals were then intubated and administered isoflurane gas anesthesia (Isovet, 1–2.5%, inhalation) alongside a potent analgesic, morphine (Morphine Aguettant, 2 mg kg^−1^, intramuscularly). After conducting an ocular examination by OCT imaging (Atlantis OCT, Topcon) to ensure the integrity of the eyes, the animals were prepared for enucleation. Local anesthesia was achieved using a procaine-based solution (Procamidor, 17.3 mg ml^−1^, 0.1 ml per eye, subcutaneously) delivered through three to four subcutaneous injections around the orbital area. After enucleation, the animals were killed using a lethal dose of pentobarbital (Dolethal; 180 mg kg^−1^, intravenously). Similarly to the preparation of human eyes, the anterior segment and vitreous body were removed immediately after enucleation, resulting in the preservation of the eyecup. Foveal retinal explants were obtained from macaque eyes that had not been treated. Peripheral retinal explants were sourced from macaques that had undergone subretinal injections, which, upon injection, caused the temporary formation of localized blebs. Regions impacted by these blebs were identified and annotated. Our collaborators provided peripheral, untreated areas of the retina for our use (excised with a 4 mm punch, Kai Medical, BPP-40F), chosen to avoid the bleb-affected zones. HD-MEA recordings were performed at two laboratories. A set of experiments was conducted directly on-site at SILABE, Mittelhausbergen, whereas another set required the transport of samples from Mittelhausbergen to Basel. The eye cups destined for Basel were submerged in pre-oxygenated (95% O_2_ and 5% CO_2_) Ames’ medium (Sigma-Aldrich, A4034) and airlifted by helicopter (Helitrans AG) to minimize the transit time. Experiments conducted on-site did not involve any sample transportation. Under both on-site and transport conditions, akin to the handling of human retinal tissue, isolated macaque retinal explants, each measuring approximately 6 mm^2^, were positioned flat on the HD-MEA for recordings. Throughout our analysis, data from both transport and nontransport conditions were processed in the same manner. Our findings revealed no differences between the two groups; therefore, they were pooled for all analyses.

### Electrophysiological recording using HD-MEAs

We employed CMOS HD-MEAs^7^ for the electrophysiological recordings of RGCs in ex vivo explants of human and nonhuman primate retina. These arrays featured a recording area of 3.85 × 2.1 mm^2^ with 26,400 electrodes, spaced at a pitch of 17.5 μm. Signal was acquired by 1,024 recording channels at a sampling rate of 20 kHz.

To assign the extracellular action potentials to individual neurons, we used an offline automatic spike sorter^11^. Briefly, electrodes recording electrical activity were grouped into local electrode groups, each comprising up to nine electrodes. The following steps were performed independently and in parallel for each group. The electrical signal from each electrode was bandpass filtered between 0.3 and 6 kHz. Spike detection occurred when the signal surpassed a predefined threshold, set at 4.2 times the standard deviation of the noise level. For each spike, the spatiotemporal waveform was extracted and saved. Spike templates corresponding to different neurons were identified through unsupervised data dimensionality reduction followed by a mean-shift clustering algorithm. Spikes were then matched to the most similar template. Since a neuron could be detected on multiple local electrode groups, duplicate neurons were detected and removed based on the similarity of their average spike waveforms and the timing of their spikes.

### Recording spontaneous spiking activity with HD-MEAs

The HD-MEAs can record from a nearly arbitrary set of 1,024 of 26,400 electrodes simultaneously. To record spiking activity on all electrodes, we split the recording into different periods, each with a different set of electrodes (‘configurations’). We included a small subset of 45 shared electrodes, which were contained in each electrode configuration. For the first configuration, the remaining electrodes were chosen randomly. In each subsequent configuration, the remaining electrodes were chosen randomly from the set of electrodes not yet included in any electrode configuration. We repeated this process across 29 different configurations. For each configuration, electrical signals were captured for 30 s, resulting in a total recording duration of approximately 16 min. This strategy allowed us to eventually capture data from the entire array (Extended Data Fig. 2b). Notably, the subset of electrodes consistently included in every configuration provided a continuous recording across all configurations, allowing offline spike sorting. For each resulting spike-sorted RGC, we calculated the average action potential waveform across the entire array by averaging the waveforms on each electrode within each configuration. For each retinal explant, this recording protocol was repeated multiple times, strategically selecting the set of shared electrodes from various regions of interest in the preparation, such as the rim of the fovea centralis and at different eccentricities within the recorded explant.

### Recording light-evoked spiking activity with HD-MEAs

Light stimuli, consisting of full-field contrast steps, were generated and controlled using Psychtoolbox in MATLAB^51^. These light stimuli were projected to the retina using a DLP LightCrafter 4710 projector (Texas Instruments) from which the magnifying optics had been removed. The light was focused onto the retina using a Nikon camera lens and a ×2.5 customized objective (Thorlabs), illuminating an area of 2.5 × 1.9 mm^2^. Specific regions of interest on the retina were identified for recording RGC light-induced spiking activity. Configurations of up to 1,024 electrodes, centered on these regions, were selected for targeted recording. A full-field contrast step stimulus, including contrast flashes, was used as a light stimulus. The stimulus consisted of the following ‘steps’ and ‘flashes’: (1) 1 s of black, (2) a single frame (1/60 s) of white (‘flash’), (3), 1 s of black (4) then a ‘step’ to 1 s of white, (5) a single frame (1/60 s) of black (‘flash’), (6) 1 s of white and (7) 0.5 s of black. The stimulus was repeated four times (trials).

### Immunohistochemistry

Post recording, the retinal explant used for electrophysiology was removed from the HD-MEA chip and immersed in 4% paraformaldehyde (PFA) for 30 min at room temperature, then washed overnight in PBS. The sample was then immersed in 30% sucrose in PBS for 2 h, followed by three cycles of freezing and thawing. For whole-mount retinae used for anatomical analysis, intact eye bulbs were fixed in 4% PFA for at least 5 days. Following fixation, tissues were rinsed thoroughly in PBS, dissected to isolate the retina and flattened by performing relaxation cuts. Due to their size, retinae were sectioned into multiple fragments, which were processed and stained individually. For both sample types, staining was performed using the same protocol unless otherwise noted. Samples were incubated in a blocking solution composed of 10% normal donkey serum (NDS; Sigma-Aldrich, S30-M), 1% BSA (Sigma-Aldrich), 0.02% Sodium Azide (NaN_3_; Sigma-Aldrich, S2002), 0.5% Triton X-100 (Sigma-Aldrich, 93443) and 1× PBS for 2 h under shaking conditions at room temperature. For antibody incubations, the same buffer was used with 3% NDS. Samples were then incubated for 5 days at room temperature under shaking conditions in primary antibody solution containing mouse anti-beta-III-tubulin (Millipore, MAB1637; 1:200). After three PBS washes, the secondary antibody solution (same buffer, 3% NDS) was applied for 2 h under shaking conditions at room temperature. For the electrophysiology-explant sample, donkey anti-mouse IgG conjugated with Alexa-405 (Thermo Fisher Scientific, A48257; 1:200) was used; for whole-mount retinae, donkey anti-mouse IgG conjugated with Alexa-488 (Thermo Fisher Scientific, A21202; 1:200) was used. Following immunostaining, all samples were washed three times in PBS and mounted on coverslips using a glycerol-based liquid mountant (ProLong Diamond Antifade Mountant, Thermo Fisher Scientific) applied directly to fluorescently labeled tissue (Fig. 1b and Extended Data Figs. 1, 2a and 6).

### Confocal microscopy

Images were captured using a Yokogawa spinning disk confocal system attached to an Olympus microscope, operated with CellSens Software by Olympus. A composite image illustrating the human RNFL shown in Extended Data Figs. 1 and 2a was created by stitching together images taken with a ×4 objective lens. For the assembly of Extended Data Fig. 6a, individual segments of the retina were imaged separately using a ×10 objective lens. The processing of these images was performed using ImageJ software (Fiji distribution), and they were seamlessly integrated into a singular image using Adobe Photoshop.

### Analysis of light responses

We conducted the analysis of neural responses to light stimulation using Python 3.8 (libraries included numpy, pandas, scipy and the electrophysiology package elephant^52^). Time-dependent firing rates, *r*(*t*), in response to each repetition of a light stimulus (‘trial’) were determined using kernel density estimation^53^ with a Gaussian kernel^54^ (Δ*t* = 10 ms, *σ* = 50 ms). The firing rates were averaged over trials and normalized by the maximum firing rate for each neuron. To assess each neuron’s responsiveness to light, we assigned a quality index (QI^55^) calculated as

$${\rm{QI}}=\frac{{\rm{Var}}{[{ < C > }_{r}]}_{t}}{{ < {\rm{Var}}{[C]}_{t} > }_{t}},$$

where the indices *r* and *t* indicated taking the expectation or calculating the variance over trials or time bins, respectively. The QI estimated the variability of the neuron’s firing rate across trials relative to the variability of the trial-averaged firing rate. Here *C* was a *T* × *R* matrix where *T* was the number of time bins and *R* was the number of trials. A QI of 1 indicated that the neuron’s mean response consistently reflected individual trial responses and tended toward 1/*R* when responses over different trials varied substantially. Neurons with a QI lower than 0.45 were omitted from further analysis.

### Clustering

Light-responsive neurons recorded in the human fovea (*n* = 711, five retinae), human periphery (*n* = 1,364, one retina) and macaque periphery (*n* = 9,385, seven retinae) were clustered separately (Fig. 2b–d) based on their light-evoked firing rates. The dimensionality of the normalized firing rate vectors corresponded to the number of time bins (*T* = 450 time bins). Before clustering, to reduce the dimensionality, we employed the nonlinear dimensionality reduction technique known as UMAP^26^ with *n*_neighbors = 10, min_dist = 0, metric = ‘Euclidean’, *n*_components_ = 2. This resulted in 2D feature vectors, each representing the mean response of each light-responsive neuron, which we could visualize in 2D UMAP coordinates. We then performed hierarchical clustering on the 2D feature vectors using the *AgglomerativeClustering* function of the Python package SKlearn^56^ (metric = ‘Euclidean’, linkage = ‘average’). To ascertain the number of clusters, we adopted the approach delineated in ref. ^57^. To determine the optimal clustering, we plotted the number of clusters against the hierarchical clustering algorithm’s merging steps, setting dataset-specific minimum element thresholds per cluster, contingent upon the size of each distinct dataset. We set a minimum of 20 cells per cluster for the human fovea and periphery, and a minimum of 100 cells per cluster for the macaque periphery. We stopped the hierarchical clustering algorithm at the merging step that produced the maximal number of clusters that fulfilled this requirement. We excluded neurons that did not belong to distinctly separated clusters from further analysis. This procedure resulted in the identification of 17 clusters in the human fovea dataset (*n* = 481), 35 clusters in the human periphery dataset (*n* = 851) and 45 clusters in the macaque periphery dataset (*n* = 6,228), that is, we deliberately split the data into many smaller clusters. This procedure ensured that firing rate vectors reflecting noisy responses or light artifacts were grouped into their own clusters, and firing rate vectors from different RGC cell types would not be erroneously grouped into the same cluster. After assessing each cluster’s mean response to the light stimulus, we manually removed those that reflected noise or artifacts. This refinement resulted in 13 clusters for the human fovea (*n* = 347), 35 clusters for the human periphery (*n* = 851) and 42 clusters for the macaque periphery (*n* = 5844). We then reevaluated the trial-averaged and normalized firing rates of the neurons that remained after the initial analysis, by applying UMAP once more, followed again by hierarchical clustering. In this second iteration, the hierarchical clustering algorithm was stopped at varying numbers of clusters (ranging between 2 and 40). To assess the quality of the clustering, we calculated a silhouette score^58^ for each potential number of clusters. The silhouette score, in conjunction with a visual inspection of the dendrogram—which visually depicts the distances between successive merges or fusions—outputted from the AgglomerativeClustering function, guided us on determining an appropriate cut-off for the number of clusters. This process resulted in five clusters for the human fovea, ten clusters for the human periphery and seven clusters for the macaque periphery. Within our datasets, we identified ON/OFF parasol/midget cells, characterized by their distinctive transient and sustained responses to light increments and decrements, respectively^21^. In the human fovea, the five clusters (*n* = 347) indicated ON transient, ON sustained, OFF transient, OFF sustained and ON sustained with elevated background activity (Fig. 2b). Transient responding cells were classified as parasol cells (Fig. 2b, clusters 4 and 5), and cells with sustained responses were classified as midget cells (Fig. 2b, clusters 1–3). In the human periphery dataset (*n* = 746), we identified and merged clusters that displayed similar behavior to midget and parasol cells, ultimately yielding four distinct clusters—ON midget, ON parasol, OFF parasol and ON–OFF cells (Fig. 2c, clusters 1–4). For the macaque periphery dataset (*n* = 4,145), we selected the four clusters that best matched the response profiles of comparable cell types (Fig. 2d). The feature vectors of individual cells were finally plotted in a 2D UMAP coordinate space, which was then rotated to position the clusters corresponding to ON cells at the top.

### Tracking propagation of spontaneous RGC action potentials

We reconstructed the average action potential (AP) waveform of each spike-sorted RGC across the microelectrode array with the method described in ‘Recording spontaneous spiking activity with HD-MEAs’. The waveform of each neuron was represented by a 3D matrix *W*(*x*, *y*, *t*), where *x* and *y* were the electrode row and column of the HD-MEA, respectively. The center-to-center electrode distance was 17.5 μm. The time coordinate *t* was defined as the number of frames at 20 kHz resolution. We visualized *W* as a movie, where each pixel represented one electrode, and color represented the voltage value at this electrode. This visualization technique (Supplementary Videos 1 and 2) enabled us to manually trace the AP’s trajectory within the video using a custom-built user interface in MATLAB. The exact moment of AP arrival at the different electrodes along its path was determined by identifying the midpoint between the minimum and maximum of the waveform at each location (Fig. 1c). The result of this analysis was an AP trajectory, that is, a set of space–time coordinates (*x*, *y*, *t*) that defined where the AP passed at what time. We excluded AP trajectories with less than three annotated space–time coordinates from further analysis. AP trajectories were smoothed and resampled using Gaussian process regression (GPR, MATLAB function fitrgp, followed by predict, significance level of 0.05; prediction type, ‘curve’). This process was conducted separately for the *x* and *y* coordinates, yielding trajectories with space–time coordinates separated in time by Δ*t* = 0.01 s. To estimate the speed of the AP propagation, we converted the two spatial coordinates (*x*, *y*) into a travel distance, *d*, by linearly integrating the distance between successive space–time coordinates along the AP trajectories. We then calculated the AP propagation speed for each RGC by a linear regression between the travel distance and travel time. We found that the speed measurements within an initial 200-μm distance to the soma were highly variable (Extended Data Fig. 3b). This large variation was likely caused by large action potential amplitudes originating near the soma at the axon initial segment and potentially by axons leaving the soma at random angles and turning towards the optic disc. We, therefore, excluded this initial part of the axonal trajectories from the regression.

### Tracking propagation of light-evoked RGC action potentials

The recordings of light-evoked RGC responses necessitated a different recording strategy, as we aimed to spike-sort—with high quality—a large number of RGCs simultaneously. Therefore, in this dataset, the average action potential waveforms could not be mapped over the entire HD-MEA but were constrained to a smaller area of the HD-MEA. We proceeded with the analysis of these datasets as described in the section titled ‘Tracking propagation of spontaneous RGC action potentials’, with the difference that the tracking was constrained to a smaller area. The neurons for which the axonal signal amplitude was insufficient for tracking were removed from the analysis, but we did not exclude axons with tracked lengths below 200 μm by default. This resulted in the tracking of 113 midget cell axons (78 ON and 35 OFF) and 29 parasol cell axons (26 ON and 3 OFF) in the foveal dataset. For the human periphery dataset, we tracked 102 ON midget and 258 parasol cells (227 ON and 31 OFF; Fig. 2e). For the macaque periphery dataset (Fig. 2f), we tracked 294 ON midget cells and 224 ON parasol cells.

### Analysis of axon trajectories and propagation speeds

We grouped the reconstructed RGC axon trajectories based on the retinal location (quadrant and the distance from the optic disc) of the explants from which they originated. This process resulted in a total of 4,758 tracked RGC axons—1,285 from human retinal explants containing the fovea centralis (10 donors; 11 explants, including foveola (*n* = 37), fovea (*n* = 1,135), parafovea (*n* = 108) and perifovea (*n* = 5)), 1,273 from human peripheral retinal explants (87 along the superior–inferior axis and 1,186 along the naso-temporal axis; 7 donors and 20 explants), 128 from macaque retinal explants containing the fovea and 2,206 from macaque peripheral retinal explants (846 along the superior–inferior axis and 1,354 along the naso-temporal axis; 11 specimens and 16 explants). The maximum lengths over which we could track AP trajectories were 1.67 mm for human fovea, 3.06 mm for human periphery, 1.96 mm for macaque fovea and 3.33 mm for macaque periphery. For explants that contained the fovea centralis, we determined the position of the fovea centralis from the electrical activity recorded with the HD-MEA. To this end, we visualized the spiking activity of the explants as images where each pixel represented an electrode and color coded the number of spikes detected at that electrode (Extended Data Fig. 2b). In these images, the ring-like structure of high RGC density around the umbo became clearly visible as a ring of high spiking activity, which allowed us to locate the position of the center of the fovea on the HD-MEA (Extended Data Fig. 2b, white arrowhead) for each foveal explant. We determined the direction of the optic disc by plotting all the AP trajectories on top of each other and observing the characteristic bending pattern. We then rotated and shifted all the AP trajectories so that the center of the fovea was at the origin and the optic disc in the direction of 0°. This procedure effectively registered all the AP trajectories from different explants containing the fovea centralis in a shared coordinate system. We quantified the relationship between the AP propagation speeds and the positions of the corresponding RGC somas with respect to the fovea. To this end, we defined the ‘RGC angular position’ as the angle formed by two lines—one connecting the location of the first space–time coordinate of the axonal trajectory with the position of the fovea centralis, and the other extending from the fovea centralis to the optic disc (that is, fovea–optic disc axis, defined as 0°). We grouped the RGC angular positions into bins of 30°. Within these bins, we computed the mean and s.e.m. of the AP propagation speeds, as illustrated in Fig. 1g (unbinned data in Extended Data Fig. 4a).

### Analysis of AP propagation speed distributions

We constructed AP propagation speed distributions of RGC axons as histograms with 50 bins, as depicted in Extended Data Fig. 5a,c, normalizing them to probability density functions. The distributions revealed a bimodal pattern, suggesting the presence of at least two distinct RGC populations. To deconvolve these populations, we fitted a Gaussian mixture model (using MATLAB’s fitgmdist function) with two components (*k* = 2; 1,000 optimization iterations) to the speed distributions of each retinal region independently (center, mid and far periphery).

### Electron microscopy sample preparation and imaging

Fixed human retinal sections (4% PFA) were rinsed once in cacodylate buffer (0.1 M, pH 7.3) for 10 min. After two additional washes in cacodylate buffer, the sections were postfixed in 1% osmium tetroxide and 0.8% potassium ferrocyanide in 0.1 M cacodylate buffer for 1 h at 4 °C. The sections were then rinsed several times in cacodylate buffer and ultrapure distilled water, and en bloc stained with 1% aqueous uranyl acetate for 1 h at 4 °C in the dark. After several wash steps in ultrapure distilled water, the sections were dehydrated in an ethanol series (30%, 50%, 75%, 96% and 100%) at 4 °C, followed by three additional washes with absolute ethanol. The sections were first washed in acetone and then finally embedded in a mixture of resin/acetone and then in pure Epon 812 resin (EMbed 812-EMS) overnight. Sections were first flat-embedded using adhesive frames (Gene Frame, 25 μl; Thermo Fisher Scientific). Polymerization was carried out for 48 h at 60 °C. Each polymerized section was then cut into a small strip. Each strip was re-embedded in Epon resin and polymerized for an additional 2 days at 60 °C. The position of the sample within the embedding was based on the orientation of the axons within the sample. We positioned the samples so that the axons were cut into cross-section. Seventy nanometer ultra-thin sections were obtained with a diamond knife, collected on copper slot grids, coated with Formvar film and a carbon layer, stained with uranyl acetate and lead citrate and observed into a Talos L120C G2 (Thermo Fisher Scientific) operated at 120 kV, equipped with a 4k × 4k Ceta CMOS camera. The SerialEM^59^ program was used for automated image acquisition of four large areas (~9,000 µm^2^) from serial sections (polygons). Polygons were all acquired at a magnification of ×8,500. It is essential to note that resin-embedding can lead to shrinkage in biological samples, potentially affecting the estimation of axonal diameters compared to those obtained in vivo or through alternative methods^60^. However, for the purpose of this study, the relative difference in axonal diameter was the relevant quantity, not the absolute diameter.

### Estimation of axon diameters in electron microscopy images

Large polygonal areas, acquired by TEM, were cropped into 3,000 × 3,000 pixel images using ImageJ software (Fiji distribution). Using Cellpose 2.0, we trained a custom segmentation model on a random subset of these images^61^. Subsequently, each TEM image was processed using this custom-trained model. The remaining errors in the output of the automatic segmentation procedure were corrected through manual curation. The segmented outlines from each image were then exported and analyzed with ImageJ (Fiji). In Fiji, we fitted the Cellpose-generated outlines with ellipses and used the lengths of the minor axes of these ellipses as the axon diameters. This was done to ensure that a tilt of an axon with respect to the imaging plane, which would elongate the outline of the axon in the direction of the tilt, would not result in a bias toward larger axon diameters.

### Measuring RNFL thickness by OCT

To assess the thickness of the RNFL (Extended Data Fig. 7b), we conducted OCT imaging using a Zeiss Cirrus HD-OCT machine. The optic disc cube 200 × 200 scan protocol was employed for imaging. RNFL thickness measurements were obtained using the device’s built-in segmentation algorithm.

### Human foveolar reaction time

All psychophysical and imaging procedures were conducted with the approval of the independent Ethics Committee of the Medical Faculty of the Rheinische Friedrich-Wilhelms-University Bonn (Lfd Nr. 294/17) and adhered to the tenets of the Declaration of Helsinki. We recorded reaction times to single-cone photo stimulation in seven participants (four females, three males; age range = 31–46 years, median age = 33 years, no compensation was offered). To ensure spatially resolved retinal photostimulation for simple reaction time (RT) measurements in humans, a custom-built AOSLO was employed. In an AOSLO, the retina and stimulus location can be resolved with subcellular resolution for precise photoreceptor-targeted psychophysical examination. Technical details of the AOSLO instrument and stimulation techniques have been described previously^62^. In brief, carefully controlled doses of 543 nm light were briefly flashed against the 840 nm, 0.85-deg field of view raster of the AOSLO to hit either a single-cone photoreceptor or a small group of cones (Extended Data Fig. 2d–e). The light distribution on the retina in the small stimulus was 1.8 µm full width at half maximum (FWHM), considering 0.03 diopter of residual defocus and 9.2 µm FWHM in the larger stimulus. The stimulus duration was 125 µs for the small and 1,126 µs for the large stimulus. The stimulus duration also dictated the total amount of stimulus power delivered to the retina, which was 0.3 nW for the small and 12 nW for the larger stimulus.

In each trial, a stimulus was randomly placed within a central subfield of the imaging raster, and the participants were instructed to report stimulus detection as quickly as possible. Due to the relatively small size of the raster (which was visible to the participants), no additional fixation target was provided. Participants exhibited normal patterns of fixational eye movements, including microsaccades, drift and tremor. Larger deviations from central fixation occurred very rarely. The randomized stimulus placement, combined with these natural eye movements, resulted in a near-normally distributed stimulus delivery location relative to the eye’s foveal center. Stimulus delivery locations were corrected for transversal chromatic offsets^63^. To avoid any adaptation or anticipation of the next stimulus delivery, a variable time interval of 0.5–1.5 s was added after the trial onset, initiated by a keyboard press of the participant. RTs were measured as the time between stimulus delivery onset, detected in the drive signal to the acousto-optic modulator by the trigger function of a fast oscilloscope (Agilent Technologies, MSO-X 3054A), and a detection response. Participants indicated stimulus detection by pressing a custom-made hardware microswitch. Millisecond resolution without temporal interference was achieved by using an Arduino microcontroller (Arduino AG), measuring the delay between the stimulus onset indicated by the oscilloscope trigger and the participant’s button press. The measured RT served then as input to a second computer running the AOSLO experiment via a MATLAB interface and saved to a log file.

Foveal RTs were measured in four females and three males (mean age = 33 ± 4 years) with no known retinal conditions. Mydriasis and cycloplegia were induced by instilling one drop of tropicamide into the lower eyelid 15 min before experimentation and subsequent redropping if necessary.

Individual stimulus positions were recovered from single AOSLO image frames and registered to a high SNR average image of the foveolar center of each participant to ensure a precise retinal stimulus localization. The high-quality retinal images were derived by spatially registering and normalizing about 150 individual AOSLO image frames by strip-wise image registration^13^. In these images, the location of each cone was semi-manually annotated to compute a 2D map of cone density. The center of the fovea (that is, zero eccentricity), was defined as the location of the CDC, which was computed as the weighted center of the 80% density isoline contour of the full density map^14^ (Extended Data Fig. 2f). Of a total of 6,200 trials, 677 (11%) had to be discarded because the foveolar image could not be registered to the foveolar center, resulting in uncertain retinal stimulus locations. An additional 344 trials (6% of the remaining 5,523) were removed because they contained implausible RTs shorter than 140 ms, most likely because of stimulus anticipation. In total, 1,021 trials (16% of 6,200) were excluded from the analysis, leaving 5,179 valid trials.

### Reaction time differences by normalization

To increase the statistical power of our analysis of reaction times (RTs) measured using AOSLO across seven participants, we initially identified and removed outlier trials (rmoutliers, MATLAB), constituting 6% of all 5,179 trials.

Following outlier removal, we normalized the data for each participant. To normalize each participant’s data (both nasal and temporal), for each participant, we subtracted the mean RT across all trials. We then computed each participant’s pooled standard deviation. We assigned the trials of each participant to two distinct regions (temporal and nasal) based on each trial’s position relative to the CDC of the respective participant. We computed the s.d. of the RTs for the nasal and temporal regions separately, yielding two s.d. per participant. For each participant, we then computed their pooled standard deviation as follows:

$${S}_{{\rm{pooled}}}=\sqrt{\frac{\left({n}_{{\rm{nasal}}}-1\right){S}_{{\rm{nasal}}}^{2}+\left({n}_{{\rm{temporal}}}-1\right){S}_{{\rm{temporal}}}^{2}}{{n}_{{\rm{nasal}}}+{n}_{{\rm{temporal}}}-2}}.$$

To normalize the RTs, we then divided each participant’s RTs by their respective pooled standard deviation. Following normalization, the data from all participants were aggregated into a single dataset, while keeping the original assignments to the temporal and nasal regions of each participant. The results of this analysis are shown in Fig. 1l.

### Reaction time differences by robust linear regression

The previous analysis yielded no significant differences between the temporal and nasal regions of the fovea (Fig. 1l). This finding may suggest that a difference existed, but our dataset was underpowered to detect it. We therefore estimated the maximal effect size that would be consistent with a failure to reject the null hypothesis given our dataset. We tested the hypothesis that the reaction time in the temporal fovea was shorter than in the nasal fovea using a robust linear regression model using *R*’s lmrob function. We used the raw reaction time data without normalization or outlier removal. This approach yielded a 90% confidence interval which included 0 ms and bounded—at 95% confidence—the effect to less than 1.0 ms and 5.6 ms for the large and small stimulus, respectively. We also conducted the analysis for each participant individually (Extended Data Fig. 2g).

### Estimating axon orientation from human retinal whole-mounts

The reconstructed image of the human whole-mount retina had a resolution of 219 pixels mm^−1^ and a size of 12k × 12k pixels, which encoded contrast in values ranging from 0 to 255. We determined the local orientation of axon bundles in a window of 201 × 201 pixels, which we moved in steps of 50 pixels over the image. We set small contrast values below 20 to a value of 0 to remove noise on the black background of the image, and we ignored windows with a median contrast value less than 20. In each window, we used the method described in ref. ^64^ to determine the bundle orientation. Briefly, the method calculates the 2D Fourier transformation of the image within the window, which decomposes the image into a set of 2D sine waves characterized by direction, spatial phase and amplitude. Low spatial frequency components usually reflect the background of the image within the window and other unwanted image features, such as uneven illumination. High-frequency components are often dominated by noise. The method, therefore, relies on a spatial bandpass filter to block those components. After filtering, the method yielded the orientation of the spatial frequency components with the highest amplitude. The orientation corresponding to this frequency component was then returned as the orientation of the axon bundles.

### Model of axonal trajectories in the human RNFL

We modeled the geometry of the human retina as a sphere of 12 mm radius and defined a point on this sphere as the origin in polar coordinates. Opposite to the origin, we removed a spherical cap from the sphere so that the ora serrata, that is, the location of the cut, was at a geodesic distance of 125° (or 26.18 mm arclength) from the origin. In our geometry, the fovea was located at (13 mm, −4 mm) and the optic disc was located at (−10 mm, 0 mm). At the location of the optic disc and fovea, we removed a spherical cap (that is, inserted a hole) in the eye of 0.6 mm and 0.2 mm radius, respectively. For numerical reasons, we found it easier to work with a 3D geometry and therefore gave the retina a thickness of 0.24 mm. This procedure defined a geometry of a spherical shell with three circular holes representing the anterior segment of the eye, the fovea and the optic disc. We constructed a 3D mesh of the resulting geometry using MATLAB’s partial differential equation toolbox. On this mesh, we solved equation (1).

1 $$D\Delta c=f$$

The parameter *D* represented the diffusivity of the retina, *c* was the unknown concentration of the substance that guides axonal growth, and *f* was a function that reflects the spatial extent of the source at the fovea, defined as follows

2 $$f\left(d\right)={e}^{-\frac{d}{{\tau }_{d}}},$$

where *d* represented the distance from the fovea and *τ*_*d*_ was a parameter that controlled how fast the strength of the source at the fovea decayed with distance from its center. Sinks and sources were furthermore defined by Dirichlet boundary conditions at the borders of the three holes—(i) for the ora serrata, *b*_OS_, (ii) for the fovea *b*_*F*_ and (iii) for the optic disc *b*_OD_. This resulted in a model with five parameters (*D*, *τ*_*d*_, *b*_OS_, *b*_*F*_ and *b*_OD_). The model was solved using MATLAB’s solvepde function. The solution defined the concentration *c* across the retina. The directional component of the spatial gradient of *c* defines the axonal directions. To calculate axonal trajectories, we used MATLAB’s stream2 function that received the axonal directions as input.

### Fitting the model to axonal orientations

We fitted the model parameters to the regions of whole-mount immunolabeled images in which we manually annotated the location of the optic disc and fovea. We then shifted, rotated and scaled the model RNFL so that the model fovea and the model optic disc coincided with those visible in the whole-mount image. In contrast to the model, which described the local directions of the axons in the range of 0°–360°, the axonal orientations calculated from the whole-mount images were defined in the range of 0°–180°. To make these two quantities (direction and orientation) comparable, we converted the model directions to orientations. This was achieved by subtracting 180° from all directions between 180° and 360°. We then fitted the model to the axonal orientation within the foveal region by minimizing the average circular distance between the modeled orientation and the local orientations of the axon bundles, which were extracted from the whole-mount image. We applied this procedure to two whole-mount immunolabeled human retinae. The fitted values for the parameters were *D* = 0.022, *τ*_*d*_ = 1.74, *b*_OS_ = 945.66, *b*_*F*_ = 929.77 and *b*_OD_ = −3.90 for the first whole mount and *D* = 0.026, *τ*_*d*_ = 1.82, *b*_OS_ = 854.37, *b*_*F*_ = 839.97 and *b*_OD_ = −4.10 for the second whole mount. The resulting *R*^2^ values of the fits amounted to 0.91 and 0.95, respectively. However, when we used the parameter values of the fit to the first whole mount to model the axon trajectories of the second whole mount, the resulting *R*^2^ value was still 0.95, emphasizing how similar the fitted parameter values were for the two different human retinae.

### Modeling RGC axonal density across the retina

The model specified the pathways of RGC axons from their soma of origin to the optic disc. It did not specify how many RGC somas were present at each retinal location. We modeled the RGC soma density using the following function:

3 $${\rho}_{{\rm{RGC}}}\left({D}_{{\rm{fov}}}\right)={\rho}_{\min}+\left(\,{\rho}_{\max}-{\rho}_{\min}\right){e}^{-\frac{\left({D}_{{\rm{fov}}}-{r}_{{\rm{fov}}}\right)}{\tau}}$$

where *ρ*_RGC_ was the RGC density at a distance *D*_fov_ from the center of the fovea; *ρ*_min_ and *ρ*_max_ were the minimal and maximal RGC densities across the retina, respectively; *τ* was a spatial scaling parameter; and *r*_fov_ was the radius of the umbo, the area in the center of the fovea devoid of RGCs. To reflect the human RGC density^30,31^, we set the parameters to *ρ*_min_ = 1,500 RGCs per mm^2^, *ρ*_max_ = 50,000 RGCs per mm^2^, *τ* = 1.43 and *r*_fov_ = 0.2 mm. We then applied Delaunay triangulation to overlay the model retina with 167,000 triangles of approximately equal size. We determined the count of RGC somas within each triangle by integrating equation (3) across the triangle surface. Subsequently, the model enabled us to estimate, for each triangle, the axonal pathway connecting the triangle’s centroid to the optic disc. Thus, each of the 167,000 axonal pathways was linked to a certain number of RGC axons, which followed this pathway closely. To ascertain the quantity of axons traversing between two closely spaced points on the retina, designated as A and B, we identified the field lines crossing the line connecting A and B and summed up the corresponding RGC axon counts.

### Calculating RGC axonal lengths at the TEM sampling locations

For each of the locations at which we measured axon diameters using TEM, we calculated the length of the axons passing through this location by using our model. To this end, we divided the retina into small triangles. We assigned the average RGC density within its area to each triangle. We then calculated three streamlines from random locations within each triangle to the optic disc, which resulted in ~170k streamlines. For each TEM location, we calculated which streamline passed the location within a 100 µm radius. We then weighted each streamline with the RGC density of its triangle of origin and calculated its length. To calculate the number of RGCs of a specific length that passed the TEM location, we computed a weighted histogram over length, taking into account the RGC density of each streamline (Extended Data Fig. 7).

### Relative light response latency of RGC responses

To estimate the response latency of midget and parasol cells to a contrast step stimulus (‘ON–OFF’ light stimulus; Fig. 2b,c) with high temporal precision, we first grouped cells based on their light response profiles. For each RGC, we recalculated firing rate profiles at higher temporal resolution using kernel density estimation with a Gaussian kernel (*σ* = 10 ms) and a fine sampling interval (Δ*t* = 0.5 ms). To identify RGCs responding robustly to the light stimulus by estimating how repeatable an RGC elicited spikes upon stimulation with the same light stimulus, we assessed trial-to-trial repeatability. Specifically, we retained only RGCs that exhibited at least one spike in each of the four trials within at least one 30-ms time window and excluded RGCs with spike trains that had no inter-spike interval above 150 ms. This filtering step reduced the foveal dataset (shown in Fig. 2b) from 347 to 233 cells and the peripheral dataset (shown in Fig. 2c) from 746 to 467 cells. We then analyzed the following two datasets separately: (1) the filtered foveal dataset (233 cells) and (2) a combined dataset of foveal and peripheral cells (700 cells). The same analytical approach was applied to both. To classify midget and parasol cells based on high-temporal-resolution firing rates, we employed an over-clustering approach using hierarchical clustering with Euclidean distance and Ward’s linkage criterion. The optimal number of clusters was determined iteratively by increasing the cluster count (*n*_clusters) from 1 to 40 and selecting the peak number of valid clusters (clusters meeting a minimum size threshold—15 cells for the foveal dataset and 30 cells for the combined dataset). The optimal number of clusters reflected a balance between cluster separation and cluster size. This process resulted in seven clusters for the foveal dataset and nine for the combined dataset. We used the foveal dataset to compare response latencies between nasal and temporal foveal RGCs using a template-based method. For each cluster, we computed trial-averaged firing rates across RGCs, smoothed them using a Savitzky–Golay filter (MATLAB smoothdata function, window = 10 ms) and used them as templates. For each foveal RGC, the cross-correlation between its firing rate profile and its cluster template was computed independently across four 500-ms time windows following the four stimulus contrast changes (ON flash, ON step, OFF flash and OFF step). The relative response latency of each RGC was defined as the time lag yielding the highest cross-correlation coefficient across the four time windows (Extended Data Fig. 10a). Most clusters were balanced between nasal and temporal regions, with only one cluster showing a majority of temporal RGCs. We employed a robust linear regression model (lmrob, R) to estimate whether temporal cells had lower response latencies than nasal cells. There was no significant difference in the response latencies between temporal and nasal cells for midget cells, parasol cells, or all cells combined. Additionally, the analysis bounded the effect (that temporal cells have lower response latencies than nasal cells) to below 1.8 ms and 6.8 ms at 95% confidence for midget and parasol cells, respectively. For the comparison between the fovea and periphery, we analyzed the combined dataset. The template-based approach was unsuitable due to highly unbalanced clusters containing predominantly foveal or peripheral cells. Instead, we estimated absolute response latencies for each RGC by determining the temporal delay between the contrast change that elicited the highest cluster-average firing rate and the individual cell’s peak firing rate. Since this method relied on single-peak estimation rather than the full response profile, it resulted in higher variability compared to the template-based method (Extended Data Fig. 10b). Cells with absolute response latencies outside the range of 10–300 ms were excluded, which reduced the dataset from 700 to 672 cells. Relative response latencies between fovea and periphery were then calculated by subtracting the median absolute response latency of peripheral cells separately for midget and parasol cells (results shown in Extended Data Fig. 10b).

### Clustering and speed analysis of foveal and peripheral RGCs

We analyzed the unfiltered combined dataset of human foveal (*n* = 347) and peripheral (*n* = 746) RGCs, as reported in Fig. 2b,c. Time-dependent firing rates, *r*(*t*), were computed in response to each repetition of the light stimulus using kernel density estimation with a Gaussian kernel (Δ*t* = 10 ms, *σ* = 50 ms). Firing rates were averaged over trials and normalized by their L2-norm for each neuron. To reduce dimensionality before clustering, we applied UMAP with parameters *n*_neighbors = 15, min_dist = 0, metric = ‘Euclidean’ and *n*_components = 2, generating 2D feature vectors representing the mean response of each neuron. These were visualized as 2D UMAP coordinates. Hierarchical clustering was performed on the feature vectors using the AgglomerativeClustering function of the Python package SKlearn (metric = ‘Euclidean’, linkage = ‘ward’). The number of clusters was determined by visually inspecting the dendrogram of the hierarchical clustering, resulting in 12 distinct clusters (Extended Data Fig. 9a–d). We then selected the neurons whose axons we could track and calculated their AP propagation speeds. We then analyzed the propagation speeds as a function of cluster and origin (fovea versus periphery; Extended Data Fig. 9e).

### Intracellular dye injections in postmortem human retinae

Single RGCs were labeled using either Vybrant DiI cell-labeling solution (1 mM in 100% ethanol; Thermo Fisher Scientific, V22885), a lipophilic, positively charged dye that integrates into cell membranes, or Lucifer yellow CH potassium salt (Thermo Fisher Scientific, L453), a hydrophilic, negatively charged dye used for intracellular labeling. Lucifer yellow was dissolved in H_2_O to prepare an 8% (wt/vol) stock solution. On the day of the experiment, fresh aliquots were prepared by sonicating the stock solution and mixing 25 μl of it with 25 μl of intracellular patch-clamp solution (120 mM K-gluconate, 6 mM KCl, 4 mM NaCl, 10 mM HEPES, 0.2 mM EGTA, 0.3 mM Tris–GTP, 2 mM Mg-ATP, 10 mM glucose; pH adjusted to 7.2 with 5 M KOH solution; all chemicals from Sigma-Aldrich). Each aliquot was filtered by using a hydrophilic PVDF membrane filter (0.22 μm pore size; Merck Millipore, SLGV004SL) and stored at 4 °C until use. Vybrant DiI was used directly from its stock solution and sonicated before back loading into patch pipettes. Human retinal explants, spanning radially from the optic disc to the ora serrata, were obtained postmortem. Each explant was placed with the RNFL facing upward on a glass slide and affixed with two platinum weights in a Petri dish lid and delicately submerged in PBS. Dye injections were conducted under an upright microscope (Olympus, BX61WI), equipped with a ×40 dip immersion objective and a digital camera (Hamamatsu digital camera; OrcaFlash 4.0, C11440). Patch pipettes were pulled from borosilicate glass with a filament (Sutter Instruments, BF150-86-10) using a micropipette puller (WZ DMZ Zeitz-Puller Universal Micropipette Micro Electrode Puller) and polished to achieve a resistance of ~30 to 40 M Ω. Each pipette was backloaded with either 4% Lucifer yellow solution or Vybrant DiI. The patch-clamp setup included a Cora V-7B head stage (Molecular Devices), a MultiClamp 700B amplifier (Molecular Devices), a DigiData 1440A digitizer (Molecular Devices) and an upright microscope. Individual RGC somas were targeted and impaled with the patch pipette tip. Dye injection was performed using Clampex software (Molecular Devices), applying a 30–40 nA current (negative for Lucifer yellow and positive for DiI) for 30–90 min. The progress of dye uptake was monitored at brief intervals using fluorescence imaging (Olympus, U-HGLGPS) to minimize photobleaching, and the injection process was stopped once fine neurites became visible. Following dye injection, retinal explants were postfixed in 4% PFA for 30 min at room temperature and subsequently washed three times with PBS. The samples were mounted on glass slides and sandwiched with cover slips using ProLong Gold Antifade Mountant (Thermo Fisher Scientific). Confocal imaging was performed within 10 days of postfixation.

### Image acquisition of labeled RGC axons

Confocal imaging was performed on fixed retinal explants mounted on glass slides with coverslips. A subset of RGCs in the explants was labeled with either Vybrant DiI or Lucifer yellow, which have distinct excitation and emission spectra. DiI is an orange–red fluorescent dye with excitation/emission peaks at ~561/600 nm, while Lucifer yellow CH, lithium salt is a green fluorescent dye with excitation/emission peaks at 428/536 nm. Imaging was conducted using a Nikon inverted Ti2 microscope equipped with a W1-SoRa spinning disk confocal system (Nikon Healthcare) and an ORCA-Fusion Digital CMOS camera (Hamamatsu, C14440-20UP). Initial low-magnification measurements, such as the distance from the cell body to the optic disc, were acquired using a ×4 objective (Nikon, MRD00045). High-resolution imaging was then performed with a ×60 oil immersion objective (Nikon, MRD01605) and Nikon type F immersion oil. In ‘SoRa mode’, the effective magnification increased to ×240, providing a size of 35.4673 pixels per micron. RGC axons were visible in multiple fields of view (FOVs). FOVs were acquired at 2,304 × 2,304 pixels. For DiI-labeled cells, excitation was performed using a 561-nm laser and an emission filter (600/52). For Lucifer yellow-labeled cells, excitation was performed using a 445-nm laser and an emission filter (525/50).

### Image preprocessing of labeled RGC axons

The resolution of the acquired z stacks was enhanced through deconvolution using Huygens Professional software (version 24.04.0p3; Scientific Volume Imaging, http://svi.nl). The deconvolved z stacks were subsequently analyzed by applying maximum intensity projection in FIJI (ImageJ) to generate 2D images. Each image was processed using Otsu’s method to apply a threshold, isolating axonal structures from the background. The resulting binary image was skeletonized to produce a 1-pixel-wide centerline representing the axonal trajectory. Sampling points were generated along the skeleton at 1-pixel intervals. At each point, a 200-pixel-long line was drawn perpendicular to the axonal trajectory. Intensity profiles were then extracted along these lines, capturing structural variations across the axonal width (Extended Data Fig. 3f). Intensity profiles and corresponding sampling point coordinates were then used for spatially resolved analysis (Extended Data Fig. 3h,k). To perform spatial measurements along the reconstructed axonal trajectory (covering several FOVs), a workflow combining Adobe Illustrator and MATLAB was developed for alignment and data processing. Overlapping FOVs were manually stitched in Illustrator using morphological landmarks as references (Extended Data Fig. 3d). A custom JavaScript script in Illustrator exported the FOV coordinates for MATLAB analysis. This allowed for transforming local measurements within each FOV into a global coordinate system. For each intensity profile measured perpendicular to the axonal trajectory, the full width at half maximum (FWHM) was computed to quantify the local axonal diameter. These measurements were used to identify varicosities along each RGC axon^65^, and only inter-varicosity segments (IVSs) were included in subsequent analyses (Extended Data Fig. 3g). Intensity profiles from IVSs were aligned. For each IVS, an average profile was computed, and individual profiles with a correlation score below 0.95 to the average (using a template-matching approach) were excluded. Following this refinement, a new average intensity profile was generated for each IVS, and the FWHM of this average profile was calculated as a measure of IVS axonal diameter (Extended Data Fig. 3g, right). Each FOV typically contained several IVSs. Diameter measurements of all IVSs along the entire axonal trajectory (across all FOVs) were computed. Each IVS diameter value was assigned a coordinate corresponding to the midpoint between successive varicosities. These measurements were then analyzed as a function of the cumulative distance from the dye injection site (Extended Data Fig. 3h,k). At increasing distance from the injection site, the SNR worsened due to poor fluorescent signals. To take this into account, each FOV was assigned an SNR. We then normalized the SNR values for each axon by defining the lowest SNR of any FOV of this axon as 0 and the maximal SNR as 1. The IVS diameter values were then weighted by the normalized SNR (Extended Data Fig. 3h–k).

### Reporting summary

Further information on research design is available in the Nature Portfolio Reporting Summary linked to this article.

## Online content

Any methods, additional references, Nature Portfolio reporting summaries, source data, extended data, supplementary information, acknowledgements, peer review information; details of author contributions and competing interests; and statements of data and code availability are available at 10.1038/s41593-025-02011-3.

## Supplementary information

Supplementary Information Supplementary Table 1.

Reporting Summary

Supplementary Video 1 Average voltage recording of an action potential of a foveal RGC reconstructed from action potentials recorded with different electrode configurations. Data was bandpass filtered and processed to optimize visualization. The color encodes the voltage at each electrode (arbitrary unit). The pixel position encodes the electrode position. Dashed line: Outline of the explant on the HD-MEA surface. Black circle: Outline of the foveola.

Supplementary Video 2 Same as Supplementary Video 1 but for a different RGC from the same preparation.

## Source data

Source Data Fig. 1 Source data.

Source Data Fig. 2 Source data.

Source Data Fig. 3 Source data.

Source Data Fig. 4 Source data.

Source Data Extended Data Fig. 1 Source data.

Source Data Extended Data Fig. 2 Source data.

Source Data Extended Data Fig. 3 Source data.

Source Data Extended Data Fig. 4 Source data.

Source Data Extended Data Fig. 5 Source data.

Source Data Extended Data Fig. 6 Source data.

Source Data Extended Data Fig. 7 Source data.

Source Data Extended Data Fig. 8 Source data.

Source Data Extended Data Fig. 9 Source data.

Source Data Extended Data Fig. 10 Source data.

## Data Availability

Raw data are available upon request from the corresponding author. Source data are provided with this paper.

## Code availability

Custom MATLAB scripts for plotting the source data are provided with this paper. The MATLAB code for preprocessing of the electrophysiological data (‘spike sorting’) is available on GitHub (https://github.com/rdiggelmann/HDsort) and usable through the SpikeInterface project (https://pypi.org/project/spikeinterface/0.12.0/). Third-party software used during the analysis is listed in the Methods section. Custom MATLAB scripts used during the analysis, and detailed in the Methods section, are available upon request.
