# Supplementary material for: Synchronization of visual perception within the human fovea
Source: Nat Neurosci. 2025 Jul 16;28(9):1959–67. doi: 10.1038/s41593-025-02011-3 (PMC12411267; doi:10.1038/s41593-025-02011-3)
Supplement: Supplementary file 1 — Supplementary Table 1. [file 41593_2025_2011_MOESM1_ESM.pdf]

nature neuroscience

Supplementary information

https://doi.org/10.1038/s41593-025-02011-3

Synchronization of visual perception within the human fovea

In the format provided by the authors and unedited

Figures	pvalue
Fig. 1g	9.68E-09
Fig. 1h	6.27E-10
Fig. 2e (fovea - midget vs parasol)	0.0060175
Fig. 2e (periphery - midget vs parasol)	0.00028941
Fig. 2e (parasol - fovea vs periphery)	1.18E-12
Fig. 2e (midget - fovea vs periphery)	1.07E-24
Fig. 2f	1.17E-10
Extended data Fig. 3b - interval 1-2	3.74E-34
Extended data Fig. 3b - interval 2-3	0.5878
Extended data Fig. 3b - interval 3-4	0.9464
Extended data Fig. 3b - interval 4-5	0.6696
Extended data Fig. 3b - interval 5-6	0.6062
Extended data Fig. 10a (midget - nasal vs temporal)	2.42E-01
Extended data Fig. 10a (parasol - nasal vs temporal)	5.24E-01
Extended data Fig. 10b (midget - fovea vs periphery)	2.0738E-12
Extended data Fig. 10b (parasol - fovea vs periphery)	1.1784E-14
