# Supplementary material for: Synchronization of visual perception within the human fovea
Source: Nat Neurosci. 2025 Jul 16;28(9):1959–67. doi: 10.1038/s41593-025-02011-3 (PMC12411267; doi:10.1038/s41593-025-02011-3)
Supplement: Supplementary file 2 — Reporting Summary [file 41593_2025_2011_MOESM2_ESM.pdf]

Corresponding author(s): Felix Franke

Last updated by author(s): May 12, 2025

Reporting Summary

Nature Portfolio wishes to improve the reproducibility of the work that we publish. This form provides structure for consistency and transparency in reporting. For further information on Nature Portfolio policies, see our Editorial Policies and the Editorial Policy Checklist.

Statistics

For all statistical analyses, confirm that the following items are present in the figure legend, table legend, main text, or Methods section.

n/a	Confirmed	
☐	☒	The exact sample size (n) for each experimental group/condition, given as a discrete number and unit of measurement
☐	☒	A statement on whether measurements were taken from distinct samples or whether the same sample was measured repeatedly
☐	☒	The statistical test(s) used AND whether they are one- or two-sided<br>*Only common tests should be described solely by name; describe more complex techniques in the Methods section.*
☒	☐	A description of all covariates tested
☐	☒	A description of any assumptions or corrections, such as tests of normality and adjustment for multiple comparisons
☐	☒	A full description of the statistical parameters including central tendency (e.g. means) or other basic estimates (e.g. regression coefficient) AND variation (e.g. standard deviation) or associated estimates of uncertainty (e.g. confidence intervals)
☐	☒	For null hypothesis testing, the test statistic (e.g. F, t, r) with confidence intervals, effect sizes, degrees of freedom and P value noted<br>*Give P values as exact values whenever suitable.*
☒	☐	For Bayesian analysis, information on the choice of priors and Markov chain Monte Carlo settings
☒	☐	For hierarchical and complex designs, identification of the appropriate level for tests and full reporting of outcomes
☒	☐	Estimates of effect sizes (e.g. Cohen's d, Pearson's r), indicating how they were calculated

Our web collection on statistics for biologists contains articles on many of the points above.

Software and code

Policy information about availability of computer code

Data collection

Custom Matlab (R2022b or earlier), Python (v 3.8 or earlier); cellSens Dimension (v 2.3), serialEM (v 4.1), Clampex (v 10.7.0).

Data analysis

Custom Matlab (R2022b or earlier) and Python (v 3.8) code, Fiji (ImageJ), custom ImageJ (v 1.54k) macros, Adobe Photoshop 2024, Adobe Illustrator 2024, R (v 4.5.0), Cellpose 2.0, R-4.5.0 (for Windows).

Custom MATLAB scripts for plotting the source data are provided with this paper. The MATLAB code for preprocessing of the electrophysiological data ('spike sorting') is available on GitHub (https://github.com/rdiggelmann/HDsort) and usable through the SpikeInterface project (https://pypi.org/project/spikeinterface/0.12.0/). Custom MATLAB scripts used during the analysis, and detailed in the methods section, are available upon request.

For manuscripts utilizing custom algorithms or software that are central to the research but not yet described in published literature, software must be made available to editors and reviewers. We strongly encourage code deposition in a community repository (e.g. GitHub). See the Nature Portfolio guidelines for submitting code & software for further information.

Data

Policy information about availability of data

All manuscripts must include a data availability statement. This statement should provide the following information, where applicable:

- Accession codes, unique identifiers, or web links for publicly available datasets
- A description of any restrictions on data availability
- For clinical datasets or third party data, please ensure that the statement adheres to our policy

Source data are provided with this paper. Raw data is available upon request from the corresponding author.

Research involving human participants, their data, or biological material

Policy information about studies with human participants or human data. See also policy information about sex, gender (identity/presentation), and sexual orientation and race, ethnicity and racism.

Reporting on sex and gender	Sex and gender were not considered in this study.
Reporting on race, ethnicity, or other socially relevant groupings	n/a
Population characteristics	Human psychophysical experiments: Total 7 subjects; 4 female, 3 male; Age range was 31 to 46 years, median age was 33 years. Informed consent was obtained from all subjects. No compensation was offered.<br><br>Human retinal tissue for ex vivo electrophysiology and anatomical analysis was obtained from postmortem donor eyes (N = 17 donors; age range: 30–80 years. Donors had no known history of retinal disease.
Recruitment	For the psychophisics experiments, participants were members of the research group. Given the relative nature of the quantity studied (relative reaction time differences), the randomness with which a retinal location was selected (driven by natural eye movements), and that neither can be known to the subjects during test, self-selection bias or other biases would have no impact on the conclusions drawn from the results.
Ethics oversight	Ethikkommission Nordwest- und Zentralschweiz EKNZ; Ethics Committee of the Medical Faculty of the Rheinische Friedrich-Wilhelms-University Bonn (Lfd-Nr. 294/17)

Note that full information on the approval of the study protocol must also be provided in the manuscript.

Field-specific reporting

Please select the one below that is the best fit for your research. If you are not sure, read the appropriate sections before making your selection.

☒ Life sciences ☐ Behavioural & social sciences ☐ Ecological, evolutionary & environmental sciences

For a reference copy of the document with all sections, see nature.com/documents/nr-reporting-summary-flat.pdf

Life sciences study design

All studies must disclose on these points even when the disclosure is negative.

Sample size	For the psychophysical experiments we initially measured 3 subjects and performed the analysis. We then performed a statistical power analysis to estimate how many more subjects were necessary to achieve robust results. No sample size calculations were performed before the other experiments. Sample sizes were chosen according to standard sample sizes in the field.
Data exclusions	No subject was excluded from the psychophysical analysis. Out of a total of 6200 trials, 677 (11%) had to be discarded because the foveolar image could not be registered to the foveolar center, resulting in uncertain retinal stimulus locations. An additional 344 trials (6% of the remaining 5523) were removed because they contained implausible RTs shorter than 140 ms, most likely because of stimulus anticipation. In total, 1021 trials (16% of 6200) were excluded from the analysis, leaving 5179 valid trials.
Replication	The psychophysical experiments included 7 replications (participants). The human foveal axon speed measurements included 11 individual explants from 10 donor eyes. For the human peripheral axon speed measurements we recorded from 20 explants from 7 donor retinae. For the light response analysis of the human retina we recorded from 5 explants (fovea), 1 explant (periphery), and 7 explants (macaque). For the macaque peripheral recordings we recorded from 16 explants from 11 specimens. For the macaque foveal recordings we recorded from 5 explants from 4 specimens. For the model fit we recorded the axon pathways from 2 donors. For the TEM imaging we recorded from 4 locations from a single donor retina. For the optical axon diameter measurements we recorded 7 axons from 3 donors.
Randomization	There was no randomization necessary/possible as the groups were defined by experimental factors, e.g. species and location from where a biological sample was isolated.

Blinding	Blinding was not possible: The psychophysical experiments required subjects trained to use the setup. The measurements were reaction time measurements to visual stimulation. The participants were therefore necessarily aware of the stimulation location, as they needed to see the stimulus.

Reporting for specific materials, systems and methods

We require information from authors about some types of materials, experimental systems and methods used in many studies. Here, indicate whether each material, system or method listed is relevant to your study. If you are not sure if a list item applies to your research, read the appropriate section before selecting a response.

Materials & experimental systems

n/a	Involved in the study	
☐	☒	Antibodies
☒	☐	Eukaryotic cell lines
☒	☐	Palaeontology and archaeology
☐	☒	Animals and other organisms
☒	☐	Clinical data
☒	☐	Dual use research of concern
☒	☐	Plants

Methods

n/a	Involved in the study	
☒	☐	ChIP-seq
☒	☐	Flow cytometry
☒	☐	MRI-based neuroimaging

Antibodies

Antibodies used	Primary antibody:<br>- mouse anti-Beta III-tubulin, Millipore, MAB1637 (1:200);<br>Secondary antibodies:<br>- donkey anti-mouse IgG conjugated with Alexa-405, Thermo Fisher Scientific, Catalog # A48257 (1:200);<br>- donkey anti-mouse IgG conjugated with Alexa-488, Thermo Fisher Scientific, Catalog # A21202 (1:200).
Validation	The commercially available mouse anti-Beta III-tubulin antibody (Millipore, MAB1637) is validated for immunohistochemistry, immunofluorescence, and Western blot. It is specific to the neuron-specific Beta III isoform and does not cross-react with glial Beta-tubulin. Species reactivity includes human, monkey, mouse, rat, bovine, sheep, pig, and avian. (https://www.sigmaaldrich.com/CH/en/product/mm/mab1637#product-documentation)

Animals and other research organisms

Policy information about studies involving animals; ARRIVE guidelines recommended for reporting animal research, and Sex and Gender in Research

Laboratory animals	Cynomolgus macaques (Macaca fascicularis); 15 animals (age: from 4 to 18)
Wild animals	No wild animals were used for this study.
Reporting on sex	Sex was not considered in the study design.
Field-collected samples	No field collected samples for used for this study.
Ethics oversight	Comité Régional d'Ethique en Matière d'Expérimentation Animale de Strasbourg and registered with the following numbers APAFIS#5716_2016061714424948_v6 (2018/08/28), APAFIS#32591_2021072914362019_v5 (2022/04/03), and APAFIS#27357-2020092811266511_v2 (2020/12/28)

Note that full information on the approval of the study protocol must also be provided in the manuscript.

Plants

Seed stocks	n/a
Novel plant genotypes	n/a
Authentication	n/a
