# Supplementary figures and images for: Synchronization of visual perception within the human fovea
Source: Nat Neurosci. 2025 Jul 16;28(9):1959–67. doi: 10.1038/s41593-025-02011-3 (PMC12411267; doi:10.1038/s41593-025-02011-3)

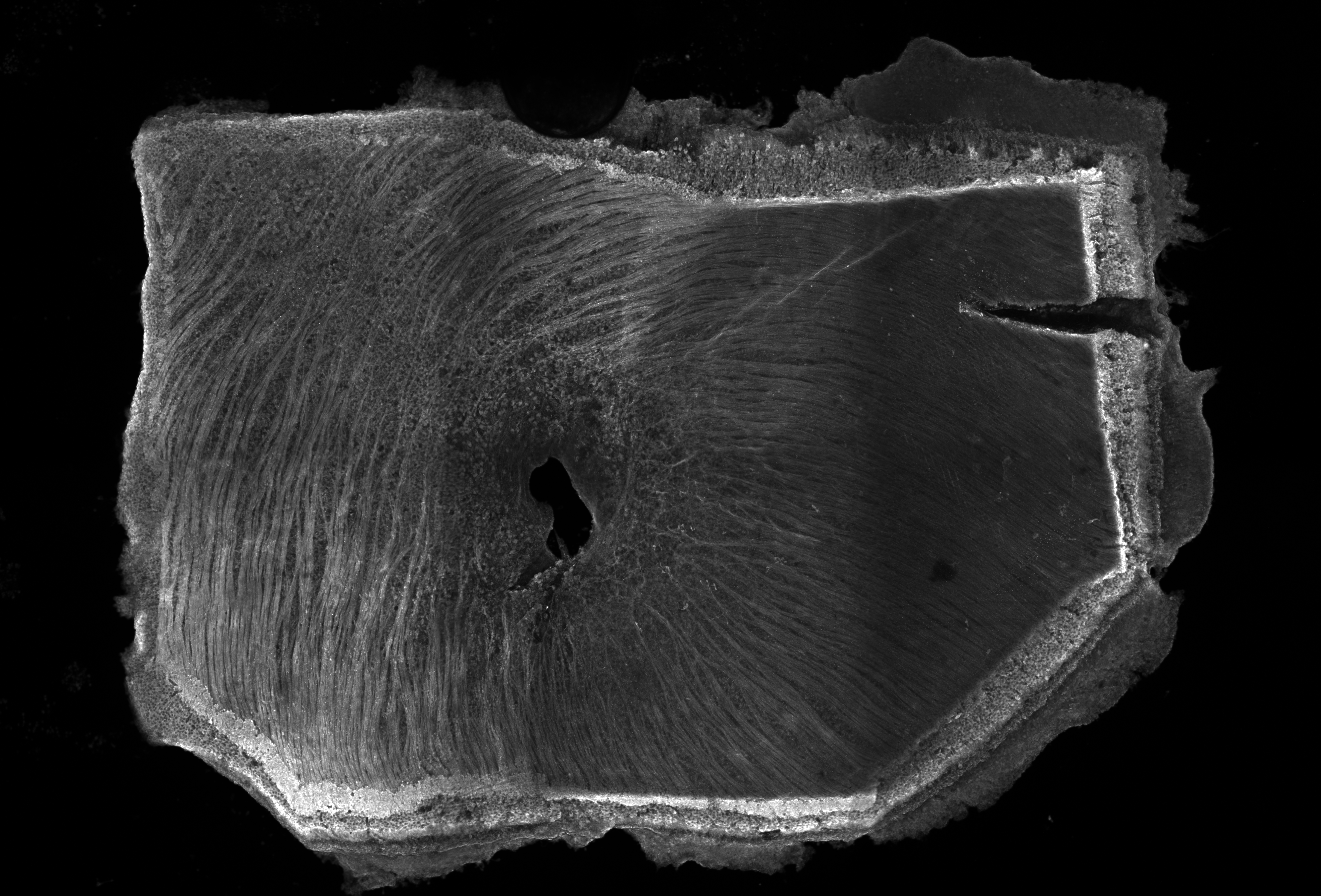

Supplement: Supplementary file 5 — Source data. [file 41593_2025_2011_MOESM5_ESM.zip › fig1/1b_b3tub_axons_fovea.tif]
